# An autoinducer-independent RhlR quorum-sensing receptor enables analysis of RhlR regulation

**DOI:** 10.1371/journal.ppat.1007820

**Published:** 2019-06-13

**Authors:** Amelia R. McCready, Jon E. Paczkowski, Jian-Ping Cong, Bonnie L. Bassler

**Affiliations:** 1 The Department of Molecular Biology, Princeton University, Princeton, New Jersey, United States of America; 2 Howard Hughes Medical Institute, Chevy Chase, Maryland, United States of America; University of Maryland, UNITED STATES

## Abstract

Quorum sensing is a chemical communication process that bacteria use to coordinate group behaviors. *Pseudomonas aeruginosa*, an opportunistic pathogen, employs multiple quorum-sensing systems to control behaviors including virulence factor production and biofilm formation. One *P*. *aeruginosa* quorum-sensing receptor, called RhlR, binds the cognate autoinducer *N*-butryl-homoserine lactone (C_4_HSL), and the RhlR:C_4_HSL complex activates transcription of target quorum-sensing genes. Here, we use a genetic screen to identify RhlR mutants that function independently of the autoinducer. The RhlR Y64F W68F V133F triple mutant, which we call RhlR*, exhibits ligand-independent activity *in vitro* and *in vivo*. RhlR* can drive wildtype biofilm formation and infection in a nematode animal model. The ability of RhlR* to properly regulate quorum-sensing-controlled genes *in vivo* depends on the quorum-sensing regulator RsaL keeping RhlR* activity in check. RhlR is known to function together with PqsE to control production of the virulence factor called pyocyanin. Likewise, RhlR* requires PqsE for pyocyanin production in planktonic cultures, however, PqsE is dispensable for RhlR*-driven pyocyanin production on surfaces. Finally, wildtype RhlR protein is not sufficiently stabilized by C_4_HSL to allow purification. However, wildtype RhlR can be stabilized by the synthetic ligand mBTL (*meta*-bromo-thiolactone) and RhlR* is stable without a ligand. These features enabled purification of the RhlR:mBTL complex and of RhlR* for *in vitro* examination of their biochemical activities. To our knowledge, this work reports the first RhlR protein purification.

## Author summary

The human pathogen *Pseudomonas aeruginosa* uses a chemical communication process called quorum sensing to orchestrate group behaviors including virulence factor production and biofilm formation. Thus, quorum sensing is essential for *P*. *aeruginosa* to be a pathogen. Quorum sensing relies on the production, release, and detection of extracellular signal molecules called autoinducers. Autoinducers are bound by partner receptor proteins, and together, autoinducer-receptor complexes control gene expression. Here, we identify, purify, and characterize a mutant version of the *P*. *aeruginosa* RhlR quorum-sensing receptor that we call RhlR*, which, remarkably, does not require its partner autoinducer to function. We show that *P*. *aeruginosa* carrying RhlR* can properly form biofilms, produce virulence factors, and infect a nematode animal used as a model for pathogenesis. Because RhlR* does not rely on an autoinducer to function, biochemical and genetic analyses that were previously not possible with RhlR could be performed with RhlR*. Indeed, our studies of RhlR* provide new insight into the workings of other *P*. *aeruginosa* quorum-sensing components, most notably, the PqsE enzyme that functions together with RhlR to control virulence factor production. We propose that RhlR* is an especially valuable tool for learning about cell-cell communication and virulence in *P*. *aeruginosa*, a pathogen of high clinical relevance.

## Introduction

Quorum sensing is a process of intercellular communication that bacteria use to coordinate group behaviors [1–4]. Quorum sensing relies on the production, release, and group-wide detection of signaling molecules called autoinducers [5–7]. At low concentrations of autoinducer, bacteria act as individuals. At high concentrations of autoinducer, bacteria act as collectives, initiating behaviors that are beneficial when undertaken in unison by the group. Many species of Gram-negative bacteria use LuxR-type quorum-sensing receptors to orchestrate group behaviors [8–10]. LuxR-type receptors are transcription factors that, as they fold, typically bind to and are stabilized and activated by cognate homoserine lactone (HSL) autoinducers [6, 11]. The opportunistic pathogen *Pseudomonas aeruginosa* employs two LuxR-type quorum-sensing receptors, LasR and RhlR, that interact with the cognate autoinducers *N*-3-oxo-dodecanoyl-L-homoserine lactone (3OC_12_HSL) and *N*-butyryl-L-homoserine lactone (C_4_HSL), respectively [8, 10]. 3OC_12_HSL and C_4_HSL are produced by the LasI and RhlI synthases, respectively. LasR activates the genes encoding RhlR and RhlI, in addition to its own regulon, so the two quorum-sensing systems function in tandem [12, 13]. RhlR also responds to a second autoinducer, designated the “alternative autoinducer”, whose identity remains unknown [14]. The alternative autoinducer is produced by PqsE [15], a thioesterase involved in alkylquinolone synthesis [16]. When bound to either C_4_HSL or the alternative autoinducer, RhlR activates transcription of many genes, including those required for virulence factor production and biofilm formation [17, 18].

LasR has been studied extensively; multiple LasR structures have been solved and the activities of wildtype and mutant LasR variants have been characterized [8, 19–22]. RhlR, by contrast, is understudied, primarily as a consequence of its biochemical intractability. The C_4_HSL autoinducer does not stabilize recombinant RhlR, which has precluded purification of the protein [23]. One possible explanation underlying these difficulties is that RhlR does not bind C_4_HSL particularly tightly, as evidenced by a micromolar EC_50_ [24]. We contrast that value to the nanomolar EC_50_ that LasR, which can be purified, exhibits for 3OC_12_HSL [21]. A synthetic ligand called *meta*-bromo-thiolactone (mBTL) has been used to successfully solubilize RhlR, enabling some preliminary analyses of activity in recombinant *E*. *coli* [23]. We use mBTL in some of the studies in the present work.

To accelerate studies of RhlR, here, we identify a RhlR mutant, RhlR Y64F W68F V133F, which we call RhlR*, that is stable and displays constitutive activity in the absence of any ligand. Threading analyses predict that all three of the RhlR* mutations reside in the ligand binding site, presumably allowing RhlR* to adopt a conformation mimicking the ligand-bound state [25]. RhlR* properly regulates quorum-sensing-controlled traits, including virulence factor production and biofilm formation. RhlR and PqsE function together to regulate the virulence factor called pyocyanin. RhlR* requires PqsE to produce pyocyanin in planktonic cultures but PqsE is dispensable for RhlR* to control pyocyanin production on surfaces. Finally, we show that the negative regulator, RsaL, prevents RhlR* from hyper-stimulating quorum-sensing-controlled genes. RhlR* represents a valuable tool for exploring RhlR structure and function.

## Results

### A screen for ligand-independent RhlR mutants

**Fig 1:**
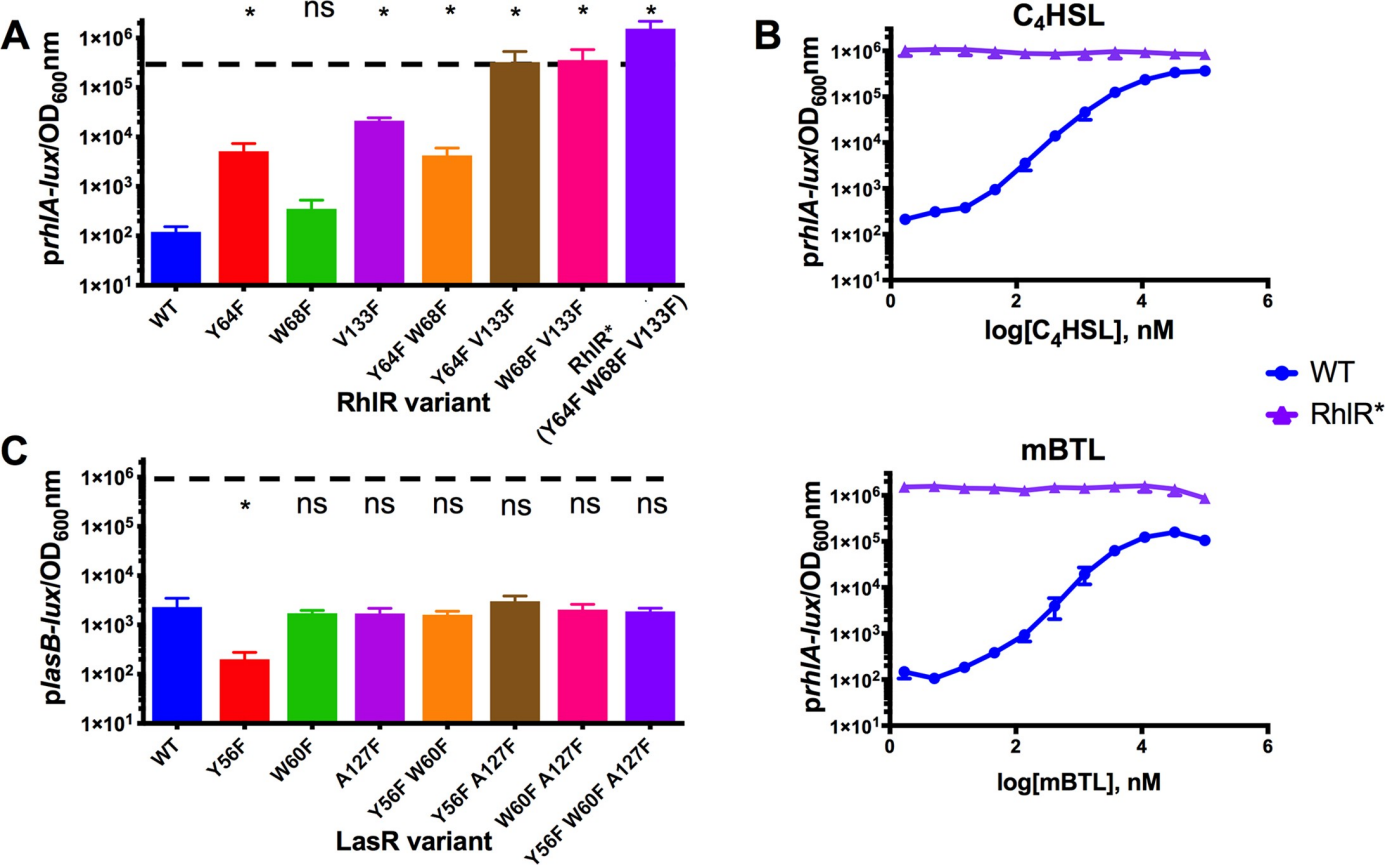
Mutational analyses of RhlR and LasR reveal ligand-independent RhlR can activate gene expression. RhlR- and LasR-controlled bioluminescence was measured in *E*. *coli*. Arabinose-inducible RhlR or LasR was produced from one plasmid and a p*rhlA-lux* (for RhlR) or p*lasB-lux* (for LasR) reporter construct was carried on a second plasmid. 0.1% arabinose was used to induce RhlR and LasR. A) Light production driven by wildtype RhlR and the designated RhlR mutants in the absence of the C_4_HSL ligand. B) RhlR-dependent bioluminescence was measured as in A for wildtype RhlR (blue) and RhlR* (purple) in response to the specified concentrations (nM) of C_4_HSL (top) and mBTL (bottom). C) Light production driven by wildtype LasR and the designated LasR mutants in the absence of the 3OC_12_HSL ligand. The color coding in panels A and C depicts the corresponding LasR and RhlR mutants. They are as follows: LasR (RhlR): Y56F (Y64F), W60F (W68F), A127F (V133F), Y56F W60F (Y64F W68F), Y56F A127F (Y64F V133F), W60F A127F (W68F V133F) and Y56F W60F A127F (Y64F W68F V133F, from here forward called RhlR*). In A and C, the dotted lines represent the maximum light produced by wildtype RhlR and LasR in response to saturating (10μM) C_4_HSL or saturating (10 nM) 3OC_12_HSL, respectively. Data show the mean of 3 biological replicates. Two technical replicates were performed and averaged for each biological replicate. Error bars represent standard error of the mean for the biological replicates. For A and C, independent *t-*tests were performed comparing each mutant to wildtype. P-values: ns ≥.05, * < .05 .

To overcome issues with RhlR biochemical intractability, we took a genetic approach to identify RhlR mutants amenable to purification that might, moreover, provide insight into the RhlR structure, ligand binding mechanism, and regulation of transcriptional activation. Toward this goal, we performed a screen for mutants of RhlR that could function in the absence of a ligand. We used our previously reported *E*. *coli* reporter assay that harbors *rhlR* on one plasmid, and a RhlR-activated p*rhlA-lux* transcriptional fusion on a second plasmid [26]. *rhlA* encodes a rhamnolipid biosynthetic enzyme required for virulence [27, 28]. The logic underlying the screen is as follows: wildtype RhlR requires its cognate autoinducer C_4_HSL for activation. Therefore, *E*. *coli* carrying wildtype *rhlR* and p*rhlA-lux* produces no light unless exogenous C_4_HSL autoinducer is provided. To identify autoinducer-independent RhlR variants, we assessed *E*. *coli* transformed with a *rhlR* mutant library for those clones that produced light in the absence of any supplied autoinducer, reasoning that such transformants must harbor RhlR variants that function independently of a ligand. We identified two RhlR mutants, RhlR Y64F and RhlR W68F, that produced 37-fold and 2.5-fold more light, respectively, than the background level produced by *E*. *coli* carrying wildtype RhlR and p*rhlA-lux* (Fig 1A). In neither case did the mutants elicit maximal luciferase activity comparable to that produced by *E*. *coli* carrying wildtype RhlR and p*rhlA-lux* in the presence of saturating (10 μM) C_4_HSL (Fig 1A, dotted line). Furthermore, increased light production occurred when C_4_HSL or mBTL was provided to the *E*. *coli* reporter strain harboring RhlR Y64F or RhlR W68F (S1 Fig). Therefore, RhlR Y64F and RhlR W68F, while harboring intrinsic autoinducer-independent activity, remain capable of ligand-driven activation.

We wondered whether a RhlR mutant could be obtained that was capable of high level transcriptional activation and was, moreover, impervious to stimulation by a ligand. One possibility was the double RhlR Y64F W68F mutant, which we constructed, but its phenotype was similar to the single RhlR Y64F mutant (Fig 1A). Thus, we sought additional mutations in RhlR that could be tested for enhancement of the ligand-independent phenotypes of the RhlR Y64F, RhlR W68F, or RhlR Y64F W68F mutants. We have previously reported that mutations at LasR A127 alter LasR responses to HSLs, and our companion structural analyses revealed that the A127 residue lies in the LasR ligand binding pocket and interacts with the autoinducer acyl tail [19, 21]. Based on protein sequence alignments and threading analyses using the LasR structure as the model, V133 is the residue in RhlR equivalent to A127 in LasR, so we explored its role in ligand-driven activation of RhlR [29]. We constructed RhlR V133F and found that, without a ligand, it stimulated 177-fold more luciferase production than wildtype RhlR in the p*rhlA-lux* assay (Fig 1A). However, in this assay, RhlR V133F did not promote emission of as much light as that elicited by wildtype RhlR in the presence of saturating ligand (Fig 1A). Combining the RhlR V133F mutation with the Y64F or W68F mutation further enhanced RhlR ligand-independent activity to approximately that made by wildtype RhlR in the presence of saturating autoinducer (Fig 1A). The RhlR Y64F W68F V133F triple mutant exhibited the highest activity of all mutants tested, exceeding the activity produced by wildtype RhlR in the presence of saturating autoinducer. Specifically, in the absence of autoinducer, the triple mutant produced approximately 10,000 times more light than did wildtype RhlR in the absence of autoinducer (Fig 1A). Provision of C_4_HSL or mBTL did not further increase the activity of RhlR Y64F W68F V133F (Figs 1B and S1). Thus, RhlR Y64F W68F V133F appears to be fully active with no ligand bound. In the remainder of this work, we refer to RhlR Y64F W68F V133F as RhlR*.

### Mutations in LasR that are homologous to those in RhlR* do not confer ligand independence to LasR.

Amino acid sequence alignments show that residues that, when altered in RhlR, confer ligand independence, are well conserved among LuxR-type receptors [30]. To investigate whether the residues we pinpointed as driving ligand-independent activity in RhlR also promote ligand-independence in LasR, we constructed the analogous set of single, double, and triple LasR mutants. These variants are: LasR Y56F, LasR W60F, LasR A127F, LasR Y56F W60F, LasR Y56F A127F, LasR W60F A127F, and LasR Y56F W60F A127F. We examined the activities of these LasR mutants along with wildtype LasR in our previously reported *E*. *coli* *lasR* p*lasB-lux* reporter system [31]. All of these mutants responded, at least partially, to exogenous 3OC_12_HSL, mBTL, or both agonists. (S2 Fig), and none of the LasR variants stimulated luciferase activity in the absence of added ligand (Fig 1C). Thus, these alterations do not confer ligand independence on LasR even though two of the residues are identical in LasR and RhlR (RhlR Y64 = LasR Y56 and RhlR W68 = LasR W60) [30]. The residue at RhlR V133 (LasR A127) is not as well conserved among LuxR family members. At this position, the residue is typically hydrophobic (A, I, L, F, M, or V) [21].

### RhlR and RhlR* protein purification and analyses

**Fig 2:**
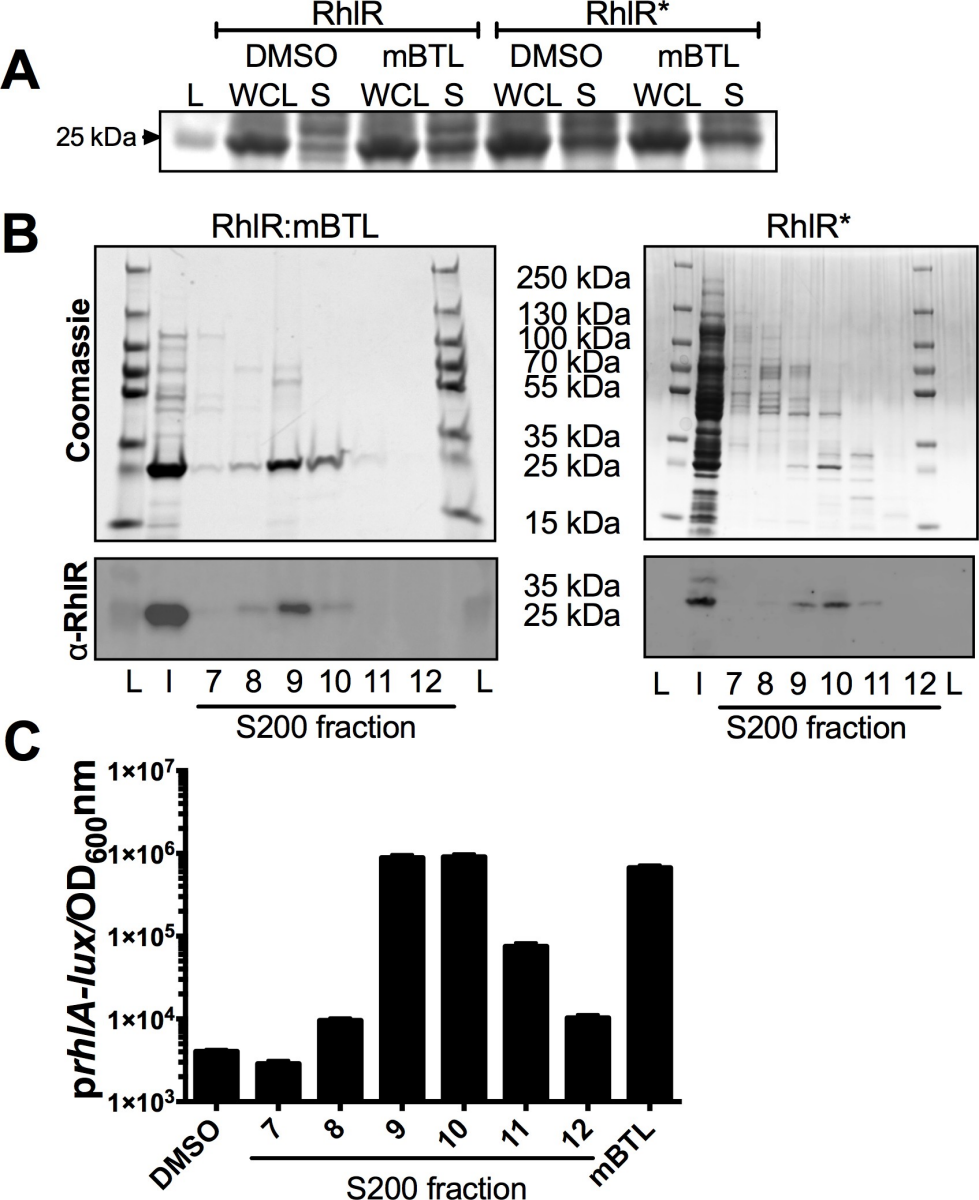
Purification of RhlR:mBTL and RhlR*. A) Total protein comparison of whole cell lysate (WCL) and soluble (S) fractions from *E*. *coli* cells overexpressing RhlR or RhlR* in the absence of ligand (DMSO) or in the presence of 100 μM mBTL. “L” denotes ladder and the 25 kDa band is designated. B) The final step in the purification of the full-length RhlR:mBTL and RhlR* proteins. Top: Coomassie stained gels with lanes loaded with 1% total volume from peak fractions 7–12 from the Superdex-200 size exclusion column. Bottom: Immunoblots of the peak fractions from the gels above using an anti-RhlR antibody [14]. “L” and “I” denote ladder and input, respectively. Molecular weight markers are designated in between the gels. C) Activity of the *E*. *coli* p*rhlA-lux* reporter strain (see Fig 1) in response to released mBTL from purified RhlR protein. The assay was carried out as described for Fig 1. As controls, 1% DMSO (no autoinducer; left-most bar) was added and pure mBTL (right-most bar) was assayed at 10 μM. The amount of mBTL released from the purified proteins in peak fractions 9 and 10 was sufficient to saturate the reporter strain, resulting in nearly equivalent levels of light production even though there were different amounts of protein in each fraction.

We characterized different RhlR* biochemical activities to learn how RhlR* functions without a ligand. First, as a proxy for folding, we compared the solubility of RhlR* to that of wildtype RhlR bound to mBTL, the only ligand known to be capable of solubilizing RhlR (Fig 2A) [23]. We expressed wildtype *rhlR* and rhlR* in *E*. *coli*. In the case of wildtype RhlR, we grew the recombinant strain in the absence and presence of 100 μM of mBTL. In the case of RhlR*, we did not add any ligand. As reported previously, RhlR was insoluble in the absence of the mBTL ligand [23] and in the presence of mBTL, a substantial portion of the RhlR protein was present in the soluble fraction (Fig 2A). RhlR* was soluble at levels comparable to that of the RhlR:mBTL complex (Fig 2A) showing that unlike wildtype RhlR, RhlR* can fold when it is not bound to a ligand. Addition of mBTL to *E*. *coli* expressing RhlR* did not enhance its solubility (Fig 2A), further indicating that RhlR* folds without a ligand.

We purified the RhlR:mBTL complex (Fig 2B shows the final fractions; S3A Fig shows the preceding purification step) using a protocol similar to one we developed for purification of LasR bound to HSLs and analogs (see Materials and Methods and [21]). We confirmed the presence of RhlR protein by immunoblotting with a RhlR-specific antibody (Fig 2B). We confirmed that mBTL was present by heating the purified RhlR:mBTL complex to 95°C to denature the protein and allow release of the ligand. We assayed the released ligand using the *E*. *coli rhlR* and p*rhlA-lux* reporter strain (Fig 2C). Released mBTL was also verified by mass spectrometry (S3B Fig). We used the identical protocol to purify RhlR* with no ligand added (Fig 2B). To our knowledge, this is the first report of the purification of a functional RhlR:ligand complex, and also, of course, the first time a RhlR variant has been purified without a ligand. The yield of RhlR* was low, and its purity in the peak fraction was approximately 70% compared to >95% purity for the wildtype RhlR:mBTL complex (Fig 2B). These issues hindered our ability to perform multiple biochemical analyses for comparison of RhlR* to RhlR:mBTL. To overcome this issue, we next purified maltose binding protein-tagged RhlR: mBTL and RhlR* (called MBP-RhlR:mBTL and MBP-RhlR*, S4A Fig). MBP-RhlR required the presence of mBTL to become soluble and enable purification, while MBP-RhlR* did not. MBP-RhlR* solubility was enhanced ~2-fold by mBTL (S4A Fig) and we return to this point below. Thus, MBP-RhlR and MBP-RhlR* are governed by principles similar to those of the native proteins, but, in the case of MBP-RhlR*, with markedly increased yield.

**Fig 3:**
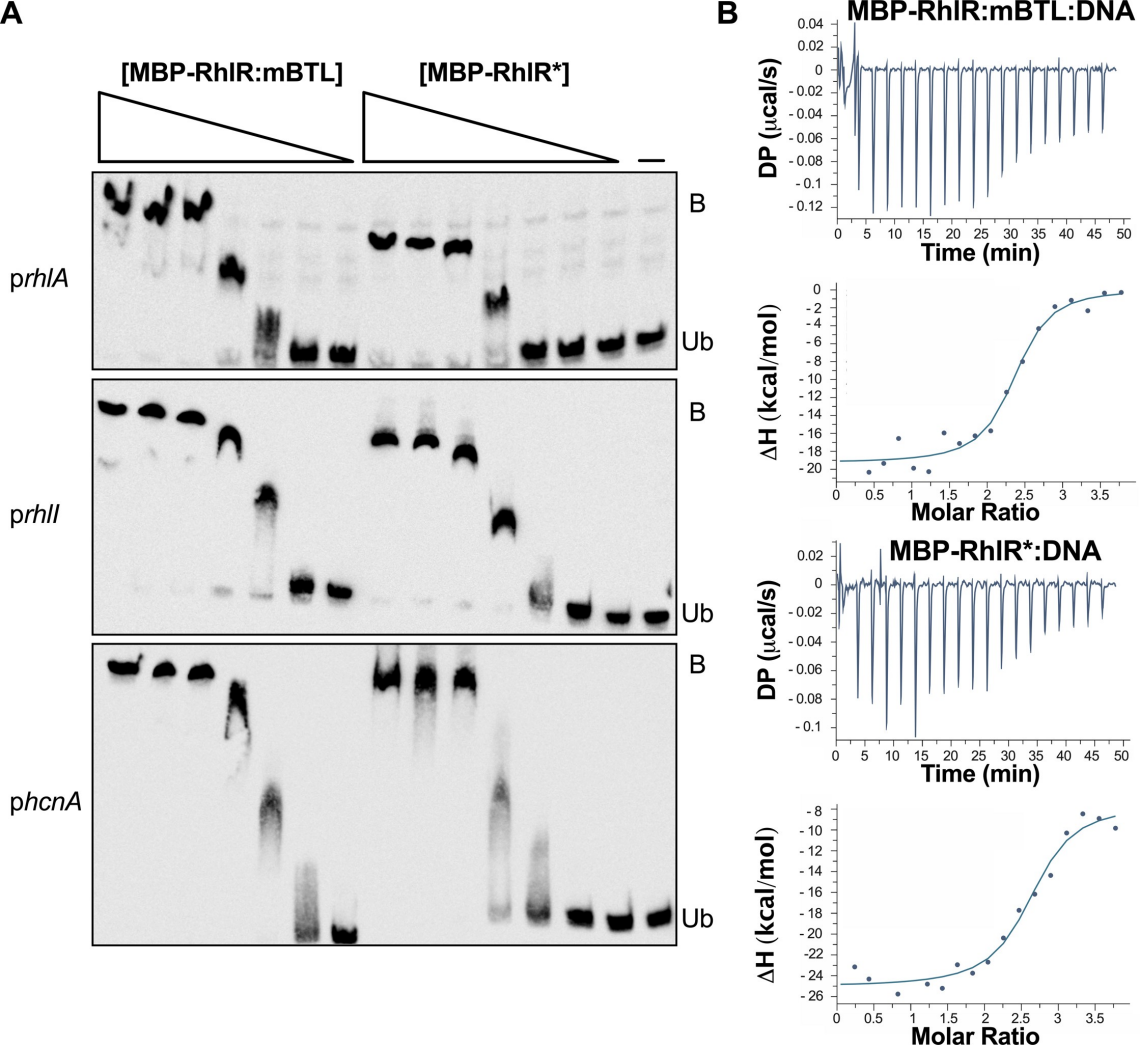
MBP-RhlR:mBTL and MBP-RhlR* bind equally well to DNA. A) Electrophoretic mobility gel shifts showing 300 bp biotin-labeled DNA fragments containing the *rhlA* (top), *rhlI* (center), and *hcnA* (bottom) promoters incubated with different concentrations of MBP-RhlR:mBTL or MBP-RhlR*. “Ub” and “B” denote unbound DNA and DNA bound to protein, respectively. The probe DNA was used at 30 ng with 500, 200, 100, 50, 30, 20, and 10 ng of the specified protein going from left to right on the gels. The right-most lane shows the no protein control (designated by the dash). B) Isothermal titration calorimetry analyses of MBP-RhlR:mBTL (top) and MBP-RhlR* (bottom) binding to the *rhl*-box consensus DNA sequence (ACCTGCCAGATTTCGCAGGT). 1 μM protein and 20 μM DNA were used in the reactions. The calculated K_d_ values for MBP-RhlR:mBTL and for MBP-RhlR* for the *rhl*-box consensus sequence are 23 nM and 34 nM, respectively. DP and ΔH denote differential power and enthalpy, respectively.

To test whether the MBP tag interfered with RhlR function, we compared the DNA binding capabilities of RhlR:mBTL and MBP-RhlR:mBTL using a gel-shift assay to assess binding to *rhlA* promoter DNA. *rhlA* expression is RhlR-dependent and the promoter contains a *rhl*-box sequence that is required for RhlR promoter binding *in vivo* (S4B Fig) [32, 33]. Both RhlR:mBTL and MBP-RhlR:mBTL bound to the *rhlA* promoter in a concentration dependent manner and, moreover, there were no differences between MBP-RhlR:mBTL and RhlR:mBTL DNA binding (S4C Fig). Addition of unlabeled competitor DNA blocked RhlR:mBTL and MBP-RhlR:mBTL binding to the labeled *rhlA* promoter (S4D Fig). These results gave us confidence that the MBP tag does not interfere with RhlR activity, and thus MBP-RhlR* would be suitable for assessment of its function. Indeed, exactly like MBP-RhlR:mBTL, MBP-RhlR* bound to the *rhlA* promoter and to two other RhlR-dependent *rhl*-box containing promoters, *rhlI* and *hcnA* (Figs 3A and S4B). Neither MBP-RhlR:mBTL nor MBP-RhlR* bound to control DNA amplified from an intergenic region of the *P*. *aeruginosa* chromosome (S4E Fig). Using isothermal titration calorimetry and the *rhl*-box consensus sequence [34], we determined the K_d_ for MBP-RhlR:mBTL and MBP-RhlR* for DNA to be 23 nM and 34 nM, respectively (Fig 3B). Thus, RhlR* functions essentially identically to RhlR bound to ligand, at least with respect to DNA binding.

RhlR* functions in our *E*. *coli* reporter assay and it can bind DNA *in vitro* with no ligand present. We thus wondered whether RhlR* can, in fact, bind a ligand. To test this possibility, we purified MBP-RhlR* in the presence of mBTL. As mentioned, MBP-RhlR* solubility was enhanced ~2-fold when mBTL was present (S4A Fig, right side). For comparison, wildtype MBP-RhlR solubility was enhanced at least 10-fold by mBTL (S4A Fig, left side). Mass spectrometry showed that mBTL was present in MBP-RhlR* when mBTL was supplied during purification (S4F Fig). Thus, while the RhlR* variant activates gene expression in a ligand-independent manner that is not enhanced by mBTL (Figs 1 and S1B), it can bind mBTL (S4A and S4F Fig).

### Ligand-independent RhlR* drives colony biofilm formation in *P*. *aeruginosa*

**Fig 4:**
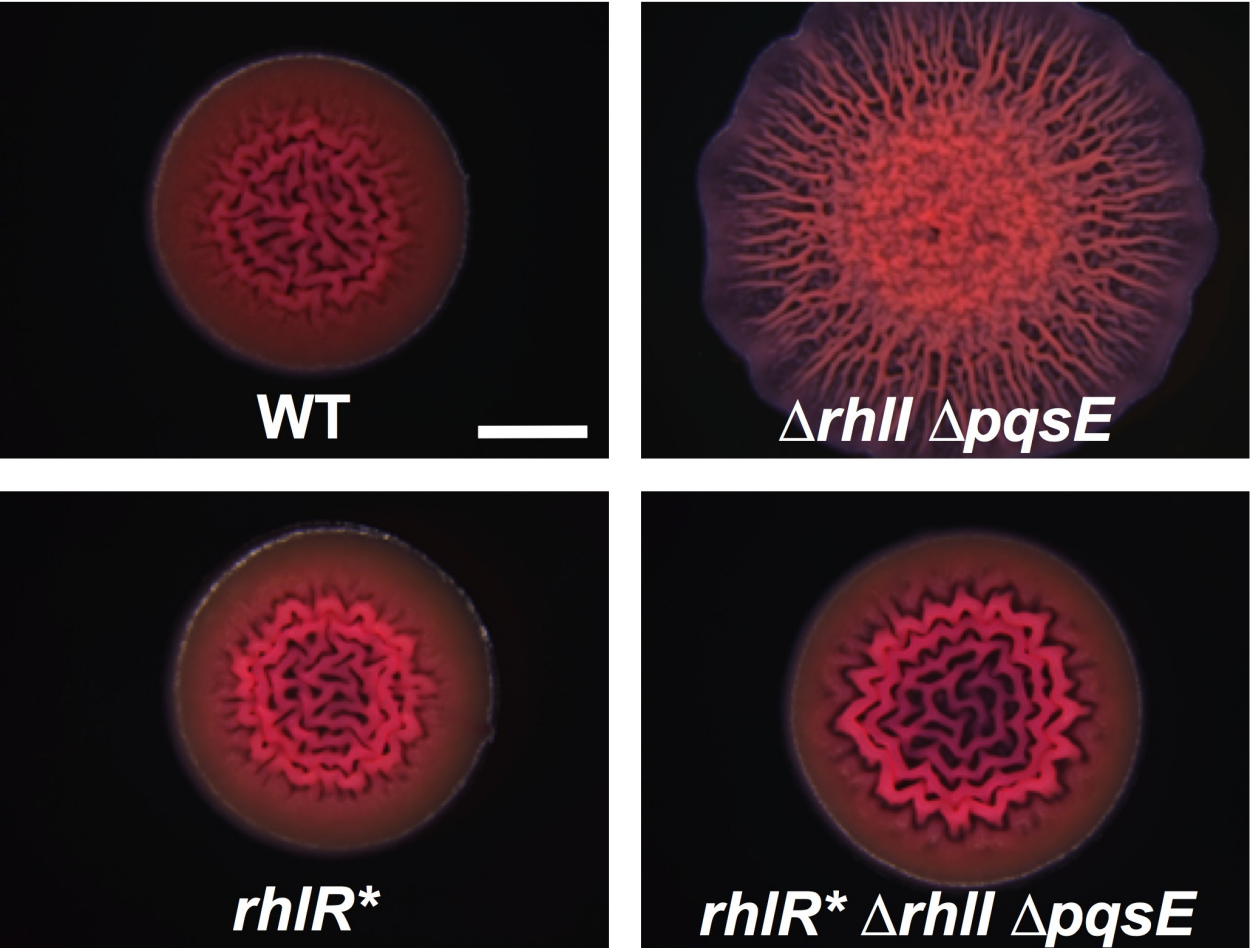
RhlR* stimulates colony biofilm formation in P. aeruginosa *in the absence of an autoinducer*. Colony biofilm phenotypes of the designated *P*. *aeruginosa* strains following growth for 120 hours on tryptone Congo-red medium. Scale bar is 2 mm for all images. Images are representative of 3 biological replicates and 2 technical replicates for each biological replicate.

Our *in vitro* analyses show that unliganded-RhlR* functions identically to wildtype RhlR bound to an autoinducer or an autoinducer mimic. To examine the consequences of RhlR* on quorum sensing *in vivo*, we engineered *P*. *aeruginosa* with rhlR* on the chromosome at its native location in an otherwise wildtype strain and in a Δ*rhlI* Δ*pqsE* strain. Our rationale for the second strain is that by deleting *rhlI* and *pqsE*, we eliminate endogenous production of both of the RhlR autoinducers; C_4_HSL (synthesized by RhlI) and the alternative autoinducer whose identity is not known (synthesized by PqsE). We measured the effect of RhlR* on colony biofilm formation in both strains. Wildtype *P*. *aeruginosa* produces colony biofilms with rugose centers and smooth peripheries on Congo red plates (Fig 4) [14, 15]. By contrast, the *P*. *aeruginosa* Δ*rhlI* Δ*pqsE* strain forms hyper-rugose colony biofilms because it is defective for phenazine production (Fig 4) [14, 15]. Both the wildtype and the Δ*rhlI* Δ*pqsE P*. *aeruginosa* strains harboring RhlR* produced colony biofilms with morphologies similar to the wildtype (Fig 4). These results suggest that, *in vivo*, RhlR* is active, ligand independent, and capable of promoting normal colony biofilm formation. Furthermore, the presence of autoinducers has no effect on this RhlR*-driven phenotype as shown in Fig 4 by the similar colony biofilms made by the rhlR* and the rhlR* Δ*rhlI* Δ*pqsE* strains.

The colony biofilm assay takes place over 120 hours. To confirm that RhlR* also functions analogously to wildtype in short time-course assays, we measured RhlR- and RhlR*-directed activation of a chromosomal p*rhlA-mNeonGreen* transcriptional fusion in Δ*rhlI P*. *aeruginosa*. We supplied DMSO or 10 μM C_4_HSL to the strains. At every time point, p*rhlA-mNeonGreen* expression in the RhlR* strain to which either DMSO or C_4_HSL had been added was within ~15% of that in the strain carrying wildtype RhlR supplied with C_4_HSL (S5 Fig). Taken together, these results show that RhlR and RhlR* are functionally equivalent for gene expression and in complex phenotypes such as colony biofilm formation.

### RhlR* requires PqsE to activate pyocyanin production in liquid

**Fig 5:**
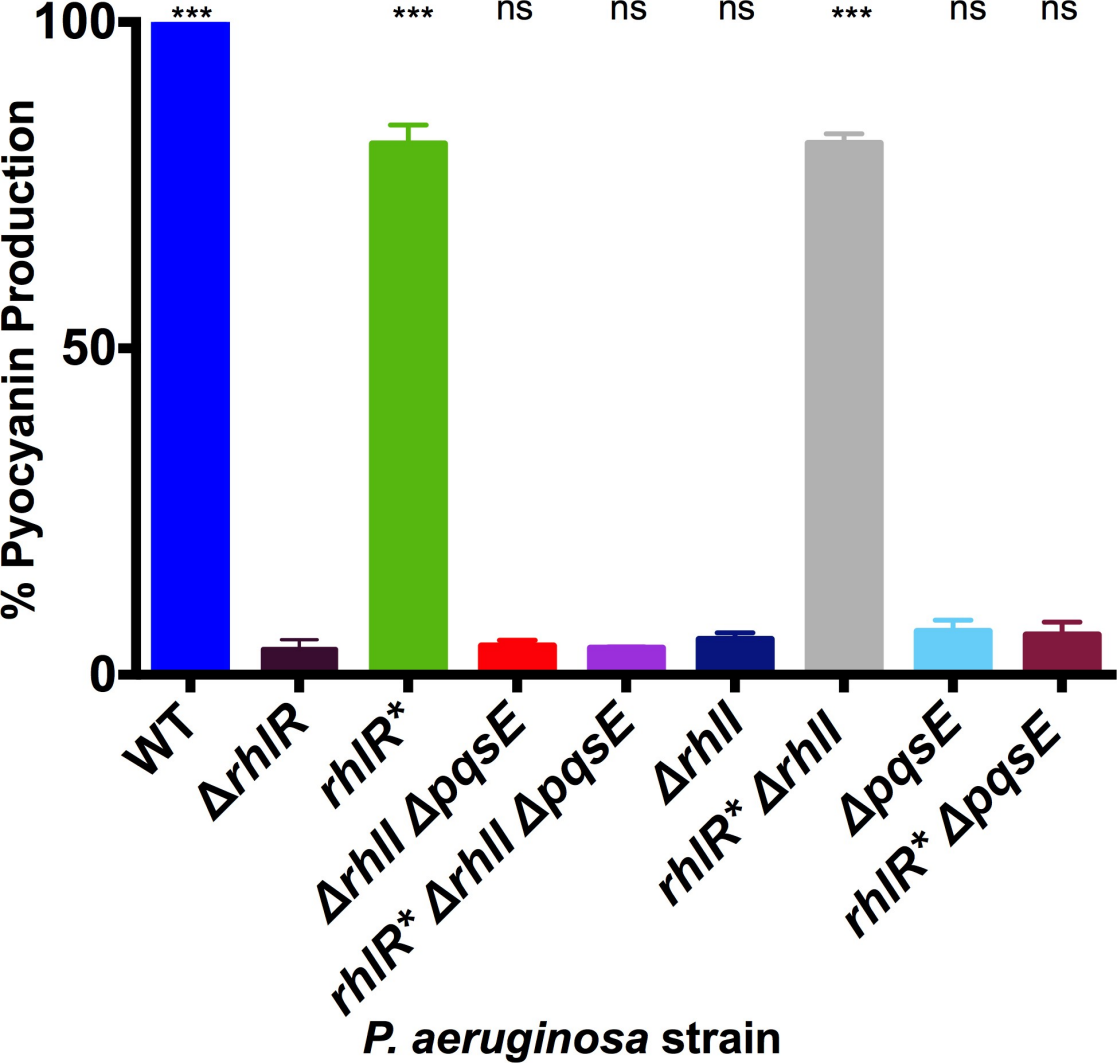
RhlR* requires PqsE but not RhlI to stimulate pyocyanin production in liquid culture. Pyocyanin production was measured in the designated *P*. *aeruginosa* strains and quantified as pigment production (OD_695_ nm) divided by cell density (OD_600_ nm). Wildtype *P*. *aeruginosa* pyocyanin production was set to 100% to make comparisons. Bars are representative of 3 biological replicates. Two technical replicates were performed and averaged for each biological replicate. Error bars represent standard error of the mean for the biological replicates. Unpaired *t-*tests were performed to compare pyocyanin production from each strain to that produced by the Δ*rhlR* strain. P-values: ns ≥ .05, *** < .001.

RhlR is a crucial regulator of *P*. *aeruginosa* virulence. Unlike wildtype RhlR, RhlR* activity is not subject to the cell-density-dependent accumulation of autoinducer. For this reason, we wondered if RhlR* could properly regulate virulence factor production. To investigate this issue, we measured pyocyanin production, which depends on RhlR [10]. Wildtype and rhlR* P. *aeruginosa* strains produced, respectively, 26-and 21-fold more pyocyanin than the Δ*rhlR* strain (Fig 5). Thus, RhlR* can substitute for RhlR to control pyocyanin production. Interestingly, the Δ*rhlI* Δ*pqsE* and rhlR* Δ*rhlI* Δ*pqsE* strains produced almost no pyocyanin (Fig 5). This result suggested that, unlike for colony biofilm formation and *rhlA* transcription, RhlR* may require an autoinducer to promote pyocyanin production. To explore this possibility, we examined the reliance of RhlR* on RhlI and on PqsE for pyocyanin production. The Δ*rhlI* strain produced almost no pyocyanin while the rhlR* Δ*rhlI* strain produced pyocyanin at a level equivalent to the rhlR* strain (Fig 5). These results show that C_4_HSL is not required for RhlR* to activate pyocyanin production. The Δ*pqsE* strain produced almost no pyocyanin, a phenotype that has been reported previously [35, 36], and likewise, the rhlR* Δ*pqsE* strain also did not make pyocyanin (Fig 5). Thus, RhlR* requires PqsE to drive pyocyanin production in this assay.

One interpretation for the above results is that, to promote pyocyanin production in liquid cultures, RhlR* requires the alternative autoinducer that is synthesized by PqsE. However, evidence already exists suggesting this cannot be the case: PqsE thioesterase activity is required for alternative autoinducer synthesis [15] but not for pyocyanin production in liquid culture [37]. An alternative interpretation is that PqsE plays a second role in the regulation of pyocyanin production in liquid beyond that of alternative autoinducer synthesis, and RhlR* cannot bypass this additional PqsE function. We offer some ideas to underpin this second hypothesis in the Discussion.

### *P*. *aeruginosa* strains harboring RhlR* are pathogenic in a *Caenorhabditis elegans* infection assay

To explore RhlR* function in the context of an animal infection model, we employed the *Caenorhabditis elegans* fast kill assay [38]. In this assay, *P*. *aeruginosa* rapidly kills *C*. *elegans* in a pyocyanin-dependent manner. The *C*. *elegans* fast kill assay steps are as follows: *P*. *aeruginosa* cells are incubated for 48 hours on petri plates, enabling them time to produce pyocyanin, after that, nematodes are added, and killing is subsequently assessed [39, 40]. To test the potential of our strains in this assay, we first preliminarily examined their pyocyanin production profiles on surfaces. Wildtype and rhlR* P. *aeruginosa* produced ample pyocyanin on plates (S6 Fig). The rhlR* Δ*rhlI* Δ*pqsE* strain also produced pyocyanin on surfaces, albeit less than the wildtype and rhlR* strains, and the Δ*rhlI* Δ*pqsE* strain did not produce detectable pyocyanin (S6 Fig and [15]). Thus, unlike in liquid culture, all of the strains containing rhlR* make pyocyanin on surfaces and therefore could have the capacity to be virulent in this nematode infection model.

**Fig 6:**
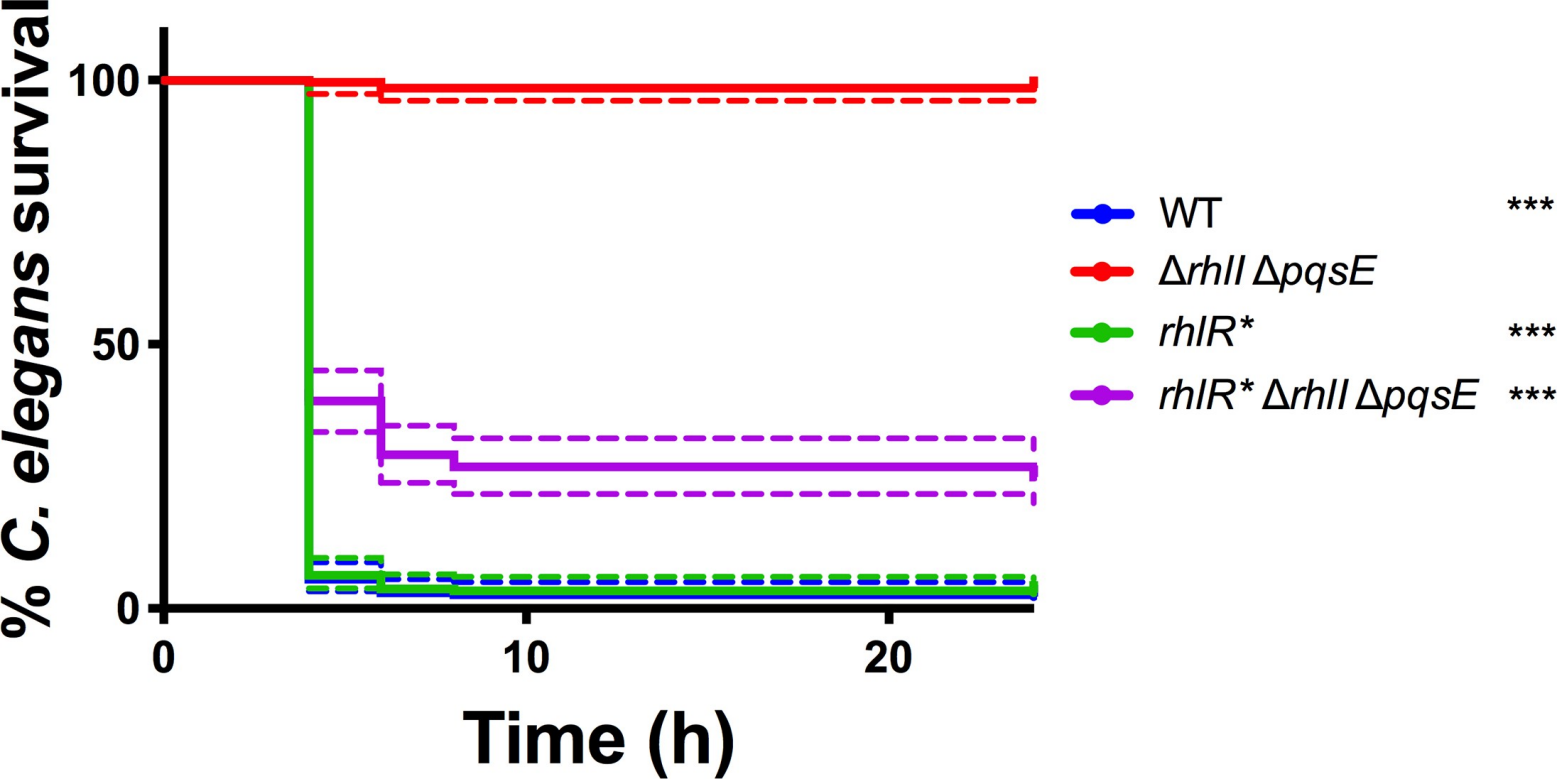
RhlR* promotes P. aeruginosa *killing of* C. elegans. Thirty age-matched L4 *C*. *elegans* nematodes were applied to lawns of the designated *P*. *aeruginosa* strains (see Materials and Methods). The percent *C*. *elegans* nematodes remaining alive were quantified on each plate. Data points represent the individuals on 9 plates (270 nematodes total for each strain) and the companion colored dotted error ranges depict 95% confidence intervals. Kaplan Meier Tests were performed to compare nematode killing by each strain to that by the Δ*rhlI* Δ*pqsE* strain. P-values: *** < .001.

In the nematode fast-kill assay, wildtype and rhlR* P. *aeruginosa* killed over 95% of the *C*. *elegans* within 24 hours (Fig 6). The rhlR* Δ*rhlI* Δ*pqsE P*. *aeruginosa* strain killed 75% of the *C*. *elegans*, consistent with our finding that the strain produces pyocyanin on surfaces, however, less than the wildtype and rhlR* strains (Figs 6 and S6). By contrast, the Δ*rhlI* Δ*pqsE* strain was highly attenuated, killing only 2% of the nematodes. (Fig 6). Again, this result tracks with the inability of this mutant to make detectable pyocyanin on plates (S6 Fig). Together, these virulence phenotypes show that RhlR* functions *in vivo* and, moreover, the phenotype of the rhlR* Δ*rhlI* Δ*pqsE* strain implies that no autoinducer is required for the strain to be highly virulent.

### RsaL prevents RhlR* from overstimulating production of quorum-sensing-controlled products

**Fig 7:**
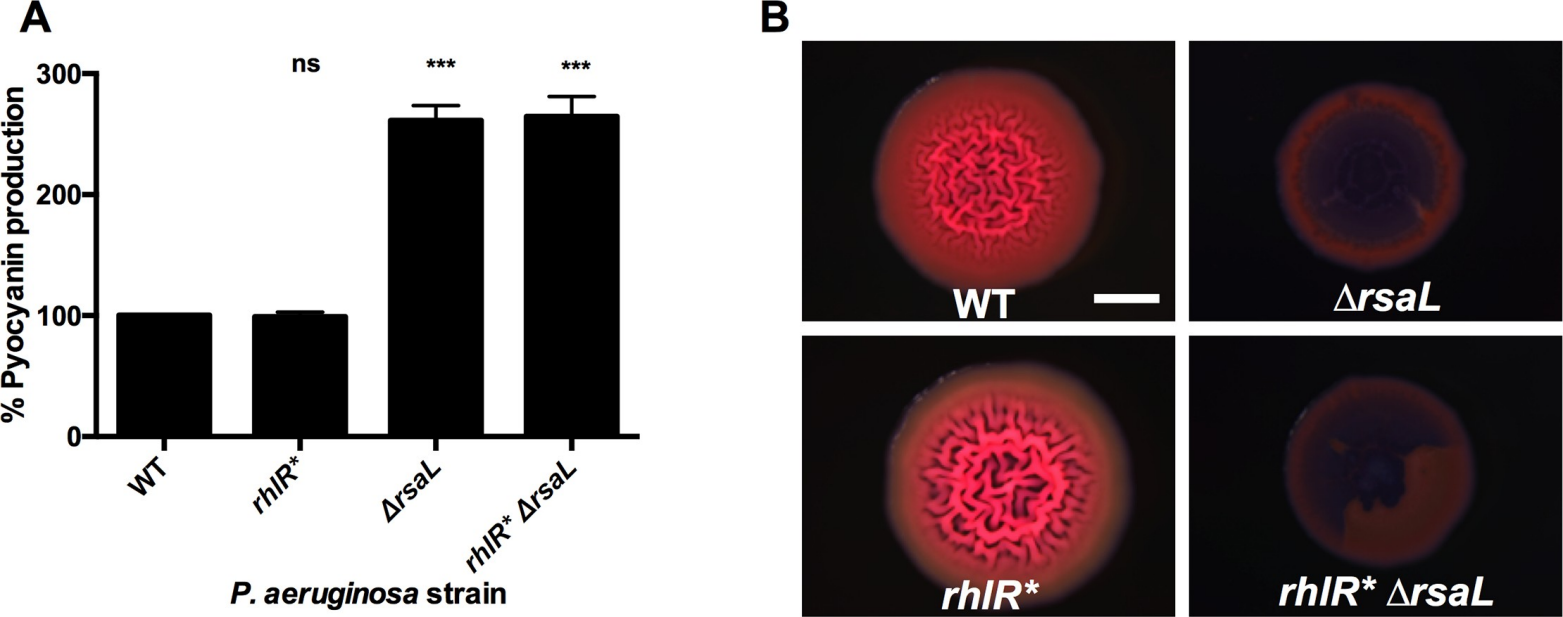
RsaL prevents RhlR* from overstimulating RhlR-controlled gene expression. A) Pyocyanin production was measured in the designated *P*. *aeruginosa* strains as in Fig 5. Production from the wildtype was set to 100%. Bars represent the means of 3 biological replicates. Two technical replicates were performed and averaged for each biological replicate. Error bars represent standard error of the mean for the biological replicates. Independent *t*-tests were performed to compare pyocyanin production from each strain to that produced by the wildtype strain. P-values are ns ≥.05, *** < .001. B) Colony biofilm phenotypes of the designated *P*. *aeruginosa* strains as in Fig 4. Scale bar is 2 mm for all images. Images are representative of 3 biological replicates and 2 technical replicates for each biological replicate.

Many RhlR-controlled products are public goods that are energetically expensive to produce. Thus, one could imagine that it would be detrimental for *P*. *aeruginosa* to harbor a ligand-independent RhlR variant that hyper-stimulates quorum-sensing-controlled gene expression. However, curiously, all of our above results show that *P*. *aeruginosa* containing RhlR* is essentially wildtype for colony biofilm formation (Fig 4), pyocyanin production (Fig 5), and virulence in nematodes (Fig 6). While our experiments do not address survival of *P*. *aeruginosa* carrying RhlR* in the wild, they nonetheless suggest that some component exists that caps RhlR* activity to normalize RhlR-controlled traits. The obvious candidate is RsaL, a transcriptional regulator that represses quorum-sensing-activated genes [38, 41]. To test this hypothesis, we measured pyocyanin production in late stationary phase (RsaL accumulates during stationary phase [42]) in wildtype, rhlR*, Δ*rsaL*, and rhlR* Δ*rsaL* strains. Both strains containing the Δ*rsaL* mutation produced significantly more pyocyanin than did the corresponding wildtype and rhlR* P. *aeruginosa* strains (Fig 7A). We also tested the role of RsaL in curbing RhlR* activity in colony biofilms. Unlike wildtype and rhlR* P. *aeruginosa*, both the Δ*rsaL* and rhlR* Δ*rsaL* strains produced colony biofilms that were smooth (Fig 7B). These colony biofilms resemble those made by the Δ*rhlI* mutant suggesting that the Δ*rsaL* and rhlR* Δ*rsaL* strains over-produce phenazines [14]. Additionally, the biofilms made by the Δ*rsaL* and rhlR* Δ*rsaL* contained voids at the centers, which we do not understand (Fig 7B). Our results show that RhlR* promotes higher pyocyanin production and drives altered colony biofilm formation in the absence of RsaL, demonstrating that RsaL prevents RhlR* from excessively stimulating quorum-sensing-controlled gene expression. Furthermore, the ligand-independent nature of RhlR* does not interfere with this RsaL regulatory role. Together, these results explain why RhlR* behaves similarly to wildtype RhlR *in vivo* and support our suggestion that RhlR* can be used as a tool to study RhlR regulation.

## Discussion

RhlR, a central component of the *P*. *aeruginosa* quorum-sensing system, controls many genes, including those required for biofilm formation and virulence factor production. Here, we report RhlR*, a constitutive RhlR allele that is stably produced and that functions without an agonist bound. There are dozens of studied LuxR-type receptors, of which RhlR is one. Almost all are unstable and inactive absent a ligand. We note that EsaR and a few other LuxR-type receptors are exceptions in that they operate by a mechanism distinct from the vast majority of LuxR-type receptors, RhlR included. Specifically, EsaR binds DNA and activates transcription when no ligand is bound and EsaR is inactive when bound to an autoinducer [43, 44]. Amongst the ligand-dependent LuxR-receptors, RhlR* represents a new type of mutant and its lack of ligand-dependence enables previously inaccessible possibilities for its study.

RhlR* contains three mutations: Y64F, W68F, and V133F. Currently, there is no RhlR crystal structure, but the locations of the RhlR* mutations provide potential insight into the RhlR structure. Based on threading, each of the RhlR* mutations resides in the putative RhlR ligand binding site and we suspect that the three hydrophobic phenylalanine residues fill and stabilize the hydrophobic ligand binding pocket, essentially mimicking the bound ligand, and in so doing, stabilize the protein [25]. This idea is supported by our previous work with LasR demonstrating that a phenylalanine substitution at LasR L130 (RhlR L136) in the LasR ligand binding pocket made the LasR L130F protein overall more stable than wildtype LasR, but not ligand independent [21]. Furthermore, mutation of the autoinducer acyl-tail binding LasR residue A127 to tryptophan prevented binding of 3OC_12_HSL and enhanced binding to HSLs possessing shorter tails suggesting that the large residue partially occupied the ligand binding site [21]. However, neither of these LasR substitutions conferred ligand-independent activity to LasR, nor did the LasR Y56F W60F A127F mutant made here (Fig 1C). We suggest that one reason RhlR may be more amenable to stabilization by bulky substitutions than is LasR is, because RhlR, to accommodate C_4_HSL, possess an intrinsically smaller binding pocket than LasR (which naturally accommodates 3OC_12_HSL), and a smaller binding pocket is more easily filled and stabilized by bulky groups.

Our analyses of *in vivo* RhlR* phenotypes show that the protein is functional with no autoinducer present, and that in this state, it can drive colony biofilm formation and nematode killing (Figs 4 and 6). The role of RhlR* in activating pyocyanin production was less straightforward concerning its reliance on PqsE (Fig 5). On surfaces, the rhlR* Δ*rhlI* Δ*pqsE P*. *aeruginosa* strain produces some pyocyanin (S6 Fig), and indeed, an amount sufficient to kill nematodes (Fig 6), so PqsE is not required for *P*. *aeruginosa* harboring RhlR* to produce pyocyanin on plates. In contrast, the *P*. aeruginosa rhlR* Δ*rhlI* Δ*pqsE* strain does not produce detectable pyocyanin in liquid culture (Fig 5), so PqsE is required. We know that one role of PqsE is in alternative autoinducer production. However, RhlR* appears to be autoinducer-independent, so we propose that PqsE must therefore have another role in pyocyanin production that RhlR* can bypass on surfaces but not in liquid culture. We do not yet understand the nature of this PqsE activity. We can imagine some possibilities: Two nearly identical but distinctly regulated *phz* (phenazine) operons, *phz1* and *phz2*, exist in *P*. *aeruginosa*. Either operon can be employed to produce the phenazine precursors required for pyocyanin biosynthesis [45, 46]. With respect to regulation, *phz2* plays the major role on surfaces while *phz1* plays a substantial role in planktonic cultures [45]. The differential regulation of these two operons mimics the differences in pyocyanin production we observe in liquid versus surface conditions. Perhaps RhlR and PqsE physically interact to activate expression of the *phz1* operon while RhlR bound to the alternative autoinducer drives expression of *phz2*. If RhlR* cannot bypass the need to interact with PqsE but can, as we have shown, function in the absence of any ligand, such a mechanism could explain the differences in pyocyanin production in our mutants on surfaces and in liquid. Alternatively, PqsE functions in production of an alkylquinolone precursor to the PQS signal molecule [16], and PQS quorum sensing is required for pyocyanin production in liquid culture [46]. Perhaps the PqsE contribution to pyocyanin production is through this alternative biosynthetic route and this pathway operates in liquid but is not required on surfaces. While further investigation is needed, our results, combined with earlier ones [15, 46], provide increasing evidence that *P*. *aeruginosa* virulence products are regulated differently under particular environmental conditions.

RhlR* behaved similarly to wildtype RhlR in all of our assays. Most surprising to us was that the high-level activation of RhlR exhibited by RhlR* did not cause increased gene expression or hyper-production of quorum-sensing-controlled products. These findings suggested to us that evolution has built checks into the *P*. *aeruginosa* quorum-sensing system that protects it against excessive stimulation. We suggest that system brakes exist both upstream and downstream of RhlR. First, the upstream brake is LasR, which is required for activation of *rhlR* expression. Thus, ligand-independent RhlR* is incapable of prematurely activating target gene expression because its own expression depends on LasR, and LasR only functions at high cell density when its cognate autoinducer, 3OC_12_HSL, has accumulated. This idea is supported by previous work demonstrating that provision of excess C_4_HSL to *P*. *aeruginosa* cultures does not prematurely activate expression of RhlR target genes [47]. Although, LasR directs the proper timing of RhlR (and RhlR*) activity, RhlR*, once made, could drive excessive activation of its target genes, but our results show this does not happen. We propose that this cap on activity is due to RsaL, that acts as a second, downstream brake on RhlR* (Fig 7). RsaL represses transcription of RhlR-activated target genes preventing their overproduction [48]. Our results combined with earlier ones [47, 48] demonstrate that while RhlR is responsible for activating a large regulon of genes in *P*. *aeruginosa*, two buffering mechanisms are present and ensure that RhlR (and RhlR*) activity is constrained to a proper window so it does not exceed the tolerable range.

Our discovery of RhlR* and its characterization provide initial insight into its biochemical activities, how it functions in conjunction with PqsE, and possibly the shape of the ligand binding pocket. The RhlR* protein offers an unparalleled opportunity for crystallization, given that wildtype RhlR and RhlR mutants reported to date have been intractable to structural analysis. Furthermore, because there is a connection between RhlR activity and PqsE activity, RhlR* could be a useful tool to discover the mechanisms underlying PqsE functions. Finally, because RhlR* is ligand-independent and active, it could be used to identify RhlR inhibitors, possibly fostering development of anti-quorum-sensing therapeutics for *P*. *aeruginosa*, fulfilling an urgent medical need.

## Materials and methods

### Mutant *rhlR* library construction

Random mutations in *rhlR* were generated using the Diversify PCR Random Mutagenesis Kit (Takara; mutagenesis level 5 protocol) with the primers ARM289 and ARM290 (S1 Table). Mutagenized DNA was digested with XhoI and SacI (NEB) and ligated into pBAD-A using T4 ligase (NEB). The resulting plasmids were transformed into One-shot TOP10 *E*. *coli* chemically competent cells (Invitrogen) along with p*rhlA-luxCDABE*. Reactions were plated on LB agar rectangular plates containing ampicillin (100 mg/L) and kanamycin (50 mg/L). Colonies were arrayed into black clear-bottom 96-well plates (Corning) using a BM3-BC colony handling robot (S&P Robotics Inc.) and screened for luciferase production. Hits were sequenced using primers ARM209 and ARM210. Candidate *rhlR* mutants were re-engineered in pBAD-A-*rhlR* using a previously reported site directed mutagenesis protocol [21]. Primers and strains used in this work are listed in S1 and S2 Tables, respectively.

### Construction of *lasR* mutants

LasR variants containing mutations homologous to those studied here in RhlR were constructed using pBAD-A carrying *lasR* and a previously reported site directed mutagenesis protocol [21]. Mutations were sequenced using primers ARM203 and ARM204 [21, 31].

### *P*. *aeruginosa* strain construction

In-frame, marker-less *rhlR* mutations were engineered onto the chromosome of *P*. *aeruginosa* PA14 as previously described [14]. Briefly, the *rhlR* gene and 500 base pairs of upstream and downstream flanking regions were cloned into pUCP18 [49]. *rhlR* mutants were constructed using pEXG2-suicide constructs with gentamicin selection and *sacB* counter selection [50, 51]. Candidate *rhlR* constructs were sequenced with *rhlR* forward and reverse primers (ARM209 and ARM210).

### *E*. *coli* p*rhlA-lux* assay for RhlR activity and p*lasB-lux* assay for LasR activity

We have previously described a method to assess RhlR activity in response to exogenous ligands, which relies on luciferase as a reporter [26]. In brief, 2 μL of overnight cultures of Top 10 *E*. *coli* carrying a plasmid harboring p*rhlA-luxCDABE* and the pBAD-A plasmid carrying either wildtype or mutant *rhlR* alleles were back diluted into 200 μL LB medium and aliquoted into clear-bottom 96-well plates (Corning). The plates were shaken at 30°C for 4 hours, at which time 0.1% arabinose was added to each well. To measure responses to a single concentration of autoinducer, either 2 μL of DMSO or 10 μM of C_4_HSL in DMSO was added to each well. To measure responses to different concentrations of ligands, ten 3-fold serial dilutions of 10 mM C_4_HSL or 10 mM mBTL were made into DMSO, and 2 μL of each dilution was added to appropriate wells. In all assays, the plates were shaken at 30°C for 4 hours. Bioluminescence and OD_600_ were measured using a Perkin Elmer Envision Multimode plate reader. Relative light units were calculated by dividing the bioluminescence measurement by the OD_600_ nm measurement. The assay to measure LasR and mutant LasR activity is identical to that for RhlR except the reporter plasmids contain *lasR* and p*lasB-luxCDABE* and 3OC_12_HSL (serially diluted from a 100 μM stock) was used as the autoinducer instead of C_4_HSL.

### RhlR production and purification

Full-length RhlR/RhlR* (cloned into pET23b) and MBP-RhlR/MBP-RhlR* (cloned into pMALC2x) were overexpressed in BL21 *E*. *coli* cells using 1 mM IPTG at 25°C for 4 hours in the presence (for wildtype RhlR) or absence (for RhlR*) of 100 μM mBTL. Cells were pelleted at 16,100 x g and resuspended in lysis buffer (500 mM NaCl, 20 mM Tris-HCl pH 8, 20 mM imidazole, 1 mM EDTA, 1 mM DTT, 5% glycerol). Resuspended cells were lysed using sonication (1 second pulses for 15 seconds). The soluble fraction from each preparation was isolated using centrifugation at 32,000 x g. For RhlR:mBTL and RhlR*, protein was prepared for heparin column binding by diluting the samples 5-fold in buffer A (20 mM Tris-HCl pH 8, 1 mM DTT). Protein was loaded on a heparin column (GE Healthcare) and eluted using a linear gradient from buffer A to buffer B (1 M NaCl, 20 mM Tris-HCl pH 8, 1 mM DTT). Peak fractions were assessed by SDS PAGE analysis and pooled. Pooled fractions were concentrated for size exclusion chromatography using a Superdex-200 (GE Healthcare) column in 200 mM NaCl, 20 mM Tris-HCl pH 8, and 1 mM DTT. Pooled fractions were concentrated to 2 mg/mL, flash frozen, and stored at -80°C. To confirm the presence of mBTL, 100 μL aliquots of fractions 7–12 from Superdex-200 columns were heated to 95°C for 15 minutes. Denatured protein was removed by centrifugation at 20,000 x g and 100 μL of the clarified supernatants were added to 900 μL of the reporter strain. For MBP-RhlR:mBTL and MBP-RhlR*, soluble fractions were applied to amylose resin (NEB) and incubated at 4°C for 1 hour. Bound protein was eluted from the resin using 10 mM maltose in lysis buffer and collected via gravity flow. Elution was repeated 10 times using 1 mL elution volumes. Fractions were pooled and concentrated. Concentrated protein was applied to a Superdex-200 column as described above.

### LC-MS/MS analysis of mBTL

600 μL of acetonitrile was added to 20 μg of RhlR:mBTL, MBP-RhlR:mBTL, or MBP-RhlR* and heated at 40°C for 1 hour to extract ligand from the protein. The sample was concentrated, and an equivalent of 2 μg was injected on an LTQ Orbitrap XL coupled to a Shimadzu HPLC (Thermo Scientific) for analysis as reported previously [52].

### Electrophoretic mobility shift assay

The *rhlA*, *rhlI*, and *hcnA* promoter sequences were amplified using PCR. The 5’-end of the forward primer for each pair was labeled with biotin (IDT). The labeled probe was incubated with 0, 10, 20, 30, 50, 100, 200, 500 ng of purified RhlR:mBTL, MBP-RhlR:mBTL, or MBP-RhlR* in binding buffer (20 mM Tris-HCl pH 8, 50 mM KCl, 1 mM EDTA, 1 mM DTT, 1.5 mg/mL poly-IC, 50 μg/mL BSA, and 10% glycerol) at room temperature for 15 minutes. DNA-protein complexes were subjected to electrophoresis on 6% DNA retardation gels (Invitrogen). DNA was visualized using the chemiluminescent nucleic acid detection module (Thermo Scientific). Briefly, DNA was transferred to a membrane, crosslinked, and incubated with blocking buffer for 15 minutes with shaking. The membranes were next incubated in stabilized streptavidin-horseradish peroxidase conjugate in blocking buffer for 15 minutes with shaking. The membranes were then washed 4 times for 5 minutes with wash buffer followed by incubation in substrate equilibration buffer for 5 minutes with shaking. Chemiluminescent substrate working solution was prepared by mixing equal parts luminol solution and stable peroxide solution. The membrane was removed from substrate equilibration buffer and incubated with the prepared chemiluminescent solution for 5 minutes without shaking. After incubation, the excess liquid was decanted and the blot was imaged on an Image Quant LAS4000 gel dock using the luminescence setting (GE Healthcare).

### Isothermal titration calorimetry

Isothermal titration calorimetry (ITC) was performed using a MicroCal PEAQ-ITC instrument (Malvern). 20 μM of *rhl*-box consensus sequence DNA (ACCTGCCAGATTTCGCAGGT) was titrated into a cell containing 1 μM of MBP-RhlR:mBTL or MBP-RhlR* at 25°C with a stirring speed of 1,000 rpm. Initial injection volume for the DNA was 0.4 μL and every subsequent injection was 2 μL. Consensus sequence DNA was resuspended in buffer to match the MBP-RhlR* and MBP-RhlR:mBTL S200 buffer. Data fitting was performed with the PEAQ-ITC Analysis software (Malvern).

### Colony biofilm assay

The protocol for this assay was adapted from [14]. In brief, 1.5 μL aliquots of overnight cultures of *P*. *aeruginosa* strains grown in 1% tryptone broth were spotted onto 60 x 15 mm plates containing 10 mL of 1% tryptone medium, 1% agar, 40 mg/L Congo red dye, and 20 mg/L Coomassie brilliant blue dye. Colony biofilms were grown for 120 hours at 25°C. Images were acquired using a Leica stereomicroscope M125 mounted with a Leica MC170 HD camera at 7.78x zoom.

### p*rhlA*-*mNeonGreen* assay

Overnight cultures of *P*. *aeruginosa* strains grown in LB medium were diluted 1:500 into 25 mL of LB medium and grown for an additional 9 hours at 37°C. One ml aliquots were collected every 1 hour starting 4 hours post-dilution. The cell density (OD_600_ nm) of each sample was measured using a Beckman Coulter DU730 Spectrophotometer. The aliquots were subjected to centrifugation at 16,100 x g for 2 minutes and the supernatants were discarded. The cell pellets were resuspended in 1 mL of PBS, and 200 μL of each resuspended sample was placed into clear-bottom 96-well plates (Corning) and fluorescence was measured using a Perkin Elmer Envision Multimode plate reader. Relative fluorescence units were calculated by dividing the fluorescence measurement by the OD_600_ nm measurement.

### Pyocyanin production assay

Overnight cultures of *P*. *aeruginosa* strains grown in LB medium were diluted 1:50 into 25 mL of LB medium and agitated for 8 hours at 37°C. 1 mL aliquots were removed from the cultures and cell density (OD_600_ nm) was measured using a Beckman Coulter DU730 Spectrophotometer. The aliquots were next subjected to centrifugation at 16,100 x g for 2 minutes and the clarified supernatants were removed and filtered through 2 μm filters. The OD_695_ nm of each supernatant was measured. Pyocyanin activity was determined by plotting the OD_695_ nm/OD_600_ nm over time for each strain. Pyocyanin assays on stationary phase cultures were performed as above, except the cultures were incubated for 24 hours prior to analysis.

### *C*. *elegans* fast kill assay

This procedure was adapted from [39]. Briefly, 10 μL aliquots from overnight cultures of the *P*. *aeruginosa* strains under study were spread onto 3.5 cm peptone-glucose-sorbitol agar medium plates (PGS) and grown for 24 hours at 37°C followed by 24 hours at 25°C. Thirty age-matched L4 *C*. *elegans* nematodes were placed onto each plate containing the bacteria. *C*. *elegans* were scored as live or dead at 4, 6, 8, and 24 hours by stroking each animal with a pick and assessing signs of movement. The percentage of live nematodes was calculated by dividing the number of live worms by the total number of worms tested for each *P*. *aeruginosa* strain and multiplying by 100. Visual inspection of pyocyanin production by *P*. *aeruginosa* strains on PGS solid medium was performed as described previously (14). In brief, strains were grown overnight in LB medium with shaking at 37°C. A 10 μL volume of each stationary phase culture was plated on PGS agar medium. Plates were incubated at 37°C for 48 hours at which time images were acquired.

## Supporting information

S1 Fig Wildtype and mutant RhlR responses to different ligands. A) RhlR-controlled bioluminescence was measured in *E*. *coli*. Arabinose-inducible RhlR was produced from one plasmid and the p*rhlA-lux* reporter construct was carried on a second plasmid. 0.1% arabinose was used for RhlR induction. Light production driven by wildtype RhlR and the designated RhlR mutants is shown in response to the specified concentrations (nM) of A) C_4_HSL and B) mBTL. Data show the means of 3 biological replicates. Two technical replicates were performed and averaged for each biological replicate. Error bars represent standard error of the mean for the biological replicates.
(TIFF)

S2 Fig Wildtype and mutant LasR responses to different ligands. LasR-controlled bioluminescence was measured in *E*. *coli*. Arabinose-inducible LasR was produced from one plasmid and the p*lasB-lux* reporter construct was carried on a second plasmid. 0.1% arabinose was used for LasR induction. Light production driven by wildtype LasR and the designated LasR mutants is shown in response to the specified concentrations (nM) of A) 3OC_12_HSL and B) mBTL. The key describing the correspondence of LasR alleles to RhlR alleles is provided in the legend to Fig 1 of the main text. Data show the means of 3 biological replicates. Two technical replicates were performed and averaged for each biological replicate. Error bars represent standard error of the mean for the biological replicates.
(TIFF)

S3 Fig Purification of RhlR:mBTL. A) Shown are the results from the initial step of purification of RhlR:mBTL by heparin chromatography. Top: UV_280_ chromatogram of the peak fractions from the heparin column. AU denotes arbitrary units. Center: Coomassie-stained gel analysis of peak fractions 24–33 (shown by the dotted lines in the top panel). 1% total volume from peak fractions was loaded in each lane. Bottom: Immunoblot of peak fractions 24–33 using an anti-RhlR antibody. Molecular weight markers are designated to the right of the gel. “L” and “I” denote ladder and input, respectively. B) Extracted ion chromatogram of 1 μM mBTL control sample (top) and the mBTL released from 2 μg of purified RhlR protein (bottom, see pooled fractions from Fig 2B of the main text). The observed and known molecular weights of mBTL are identical.
(TIFF)

S4 Fig Purification and characterization of MBP-RhlR:mBTL and MBP-RhlR*. A) The soluble fractions from lysed *E*. *coli* cells expressing MBP-RhlR or MBP-RhlR* that had been grown in the presence or absence of mBTL were incubated, in bulk, with amylose resin and eluted with 10 mM maltose in lysis buffer (see Materials and Methods). Seven 1 mL fractions were collected and 1% of the total volume of each fraction was subjected to SDS PAGE analysis. “L” denotes ladder and the 70 kDa band is designated. B) DNA sequences from -300 to -1 bp of the *rhlA*, *rhlI*, and *hcnA* promoters. Red sequences show the *rhl*-boxes. C) Electrophoretic mobility gel shift showing the 300 bp biotin-labeled *rhlA* promoter sequence incubated with decreasing concentrations of RhlR:mBTL and MBP-RhlR:mBTL. “Ub” and “B” denote unbound DNA and DNA bound to protein, respectively. The probe DNA was used at 30 ng with 500, 200, 100, 50, 30, 20, and 10 ng of the specified protein going from left to right on the gel. The right-most lane shows the no protein control (designated by the dash). D) Electrophoretic mobility gel shift showing the 300 bp *rhlA* promoter sequence labeled with biotin with or without the identical unlabeled competitor DNA. Lanes are as follows: 1) unlabeled competitor DNA alone, 2) labeled DNA alone, 3) labeled DNA and unlabeled competitor DNA, 4) labeled DNA and RhlR:mBTL, 5) labeled DNA, RhlR:mBTL, and unlabeled competitor DNA, 6) labeled DNA and MBP-RhlR:mBTL, and 7) labeled DNA, MBP-RhlR:mBTL, and unlabeled competitor DNA. The unbound biotin-labeled DNA band spreads out when it is combined with the 100-fold excess unbound unlabeled competitor DNA. This feature makes the unbound band appear thicker than when no competitor DNA is present. “Ub” and “B” denote unbound DNA and DNA bound to protein, respectively. The labeled probe DNA was used at 30 ng and unlabeled probe DNA was used in 100-fold excess. 200 ng of the different proteins were used. E) Electrophoretic mobility gel shift showing a biotin-labeled 300 bp fragment of intergenic control DNA with different concentrations of MBP-RhlR:mBTL and MBP-RhlR*. “Ub” denotes unbound DNA. The probe DNA was used at 30 ng with 500, 200, 100, 50, 30, 20, and 10 ng of the specified protein going from left to right on the gel. The right-most lane shows the no protein control (designated by the dash). F) Extracted ion chromatogram of ligand released from purified MBP-RhlR* that was produced in *E*. *coli* grown in the presence (top) or absence (bottom) of mBTL. The observed and known molecular weights of mBTL are identical.
(TIFF)

S5 Fig Comparison of RhlR- and RhlR*-dependent gene expression timing and strength. Shown are 9 h time-courses of RhlR- and RhlR*-dependent activation of expression of a p*rhlA-mNeonGreen* transcriptional fusion in Δ*rhlI P*. *aeruginosa* in the presence of 10 μM C_4_HSL or 1% DMSO. Data depict the mean of 3 biological replicates. Two technical replicates were performed for each biological replicate. Error bars depict the standard error of the mean of the biological replicates.
(TIFF)

S6 Fig The P. aeruginosa rhlR* ΔrhlI ΔpqsE strain produces pyocyanin on surfaces. Pyocyanin production phenotypes of the designated *P*. *aeruginosa* strains are shown following growth for 48 hours on PGS medium. A 10 μL aliquot of stationary phase *P*. *aeruginosa* was spotted onto each plate and the sample was spread evenly over the entire agar surface. There were no *C*. *elegans* added to the plates. Red coloration is indicative of pyocyanin production. Images are representative of 3 biological replicates and 2 technical replicates for each biological replicate.
(TIFF)

S1 Table Primers used in this study. This table lists the names, sequences (5’ to 3’), and uses of all primers employed in this study.
(ZIP)

S2 Table Strains used in this study. This table lists the names, sources, and genotypes of all strains used in this study.
(ZIP)
