# Supplementary figures and images for: An autoinducer-independent RhlR quorum-sensing receptor enables analysis of RhlR regulation
Source: PLoS Pathog. 2019 Jun 13;15(6):e1007820. doi: 10.1371/journal.ppat.1007820 (PMC6564026; doi:10.1371/journal.ppat.1007820)

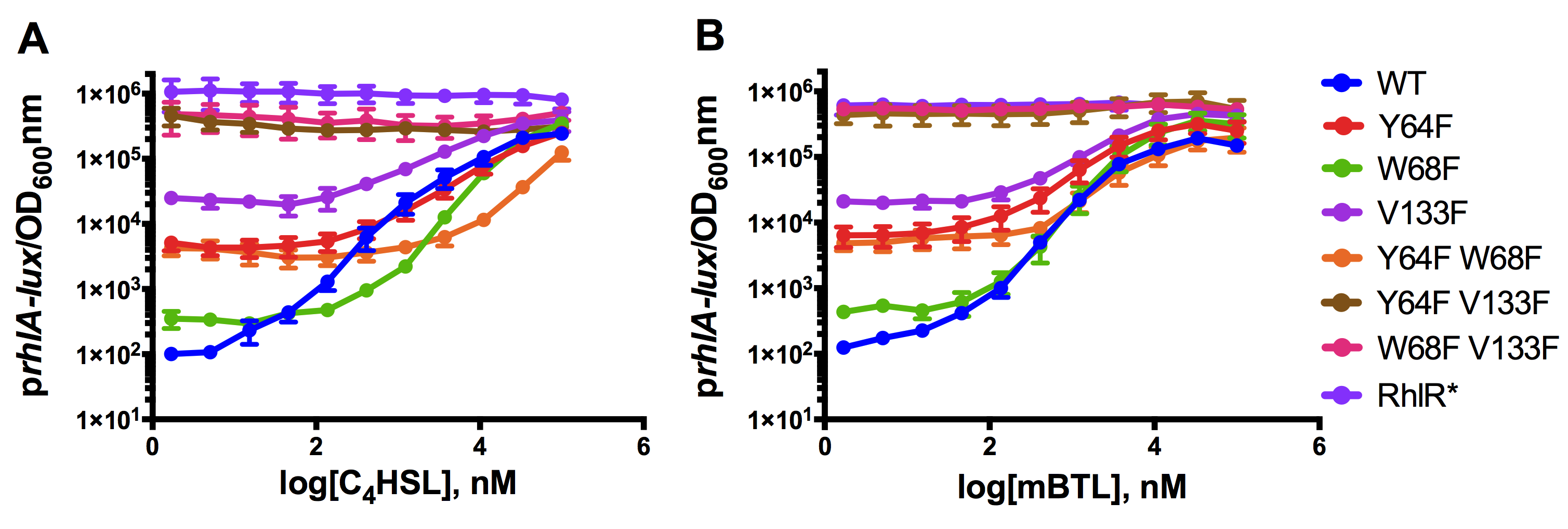

Supplement: S1 Fig — A) RhlR-controlled bioluminescence was measured in E. coli. Arabinose-inducible RhlR was produced from one plasmid and the prhlA-lux reporter construct was carried on a second plasmid. 0.1% arabinose was used for RhlR induction. Light production driven by wildtype RhlR and the designated RhlR mutants is shown in response to the specified concentrations (nM) of A) C4HSL and B) mBTL. Data show the means of 3 biological replicates. Two technical replicates were performed and averaged for each biological replicate. Error bars represent standard error of the mean for the biological replicates. (TIFF) [file ppat.1007820.s001.tiff]

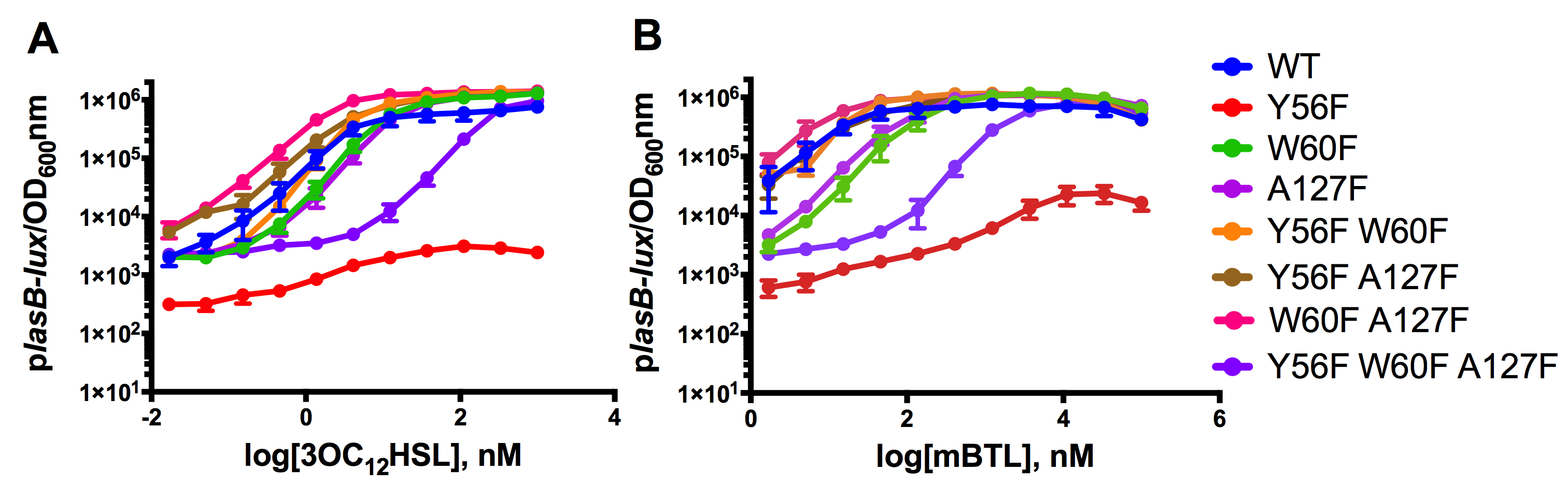

Supplement: S2 Fig — LasR-controlled bioluminescence was measured in E. coli. Arabinose-inducible LasR was produced from one plasmid and the plasB-lux reporter construct was carried on a second plasmid. 0.1% arabinose was used for LasR induction. Light production driven by wildtype LasR and the designated LasR mutants is shown in response to the specified concentrations (nM) of A) 3OC12HSL and B) mBTL. The key describing the correspondence of LasR alleles to RhlR alleles is provided in the legend to Fig 1 of the main text. Data show the means of 3 biological replicates. Two technical replicates were performed and averaged for each biological replicate. Error bars represent standard error of the mean for the biological replicates. (TIFF) [file ppat.1007820.s002.tiff]

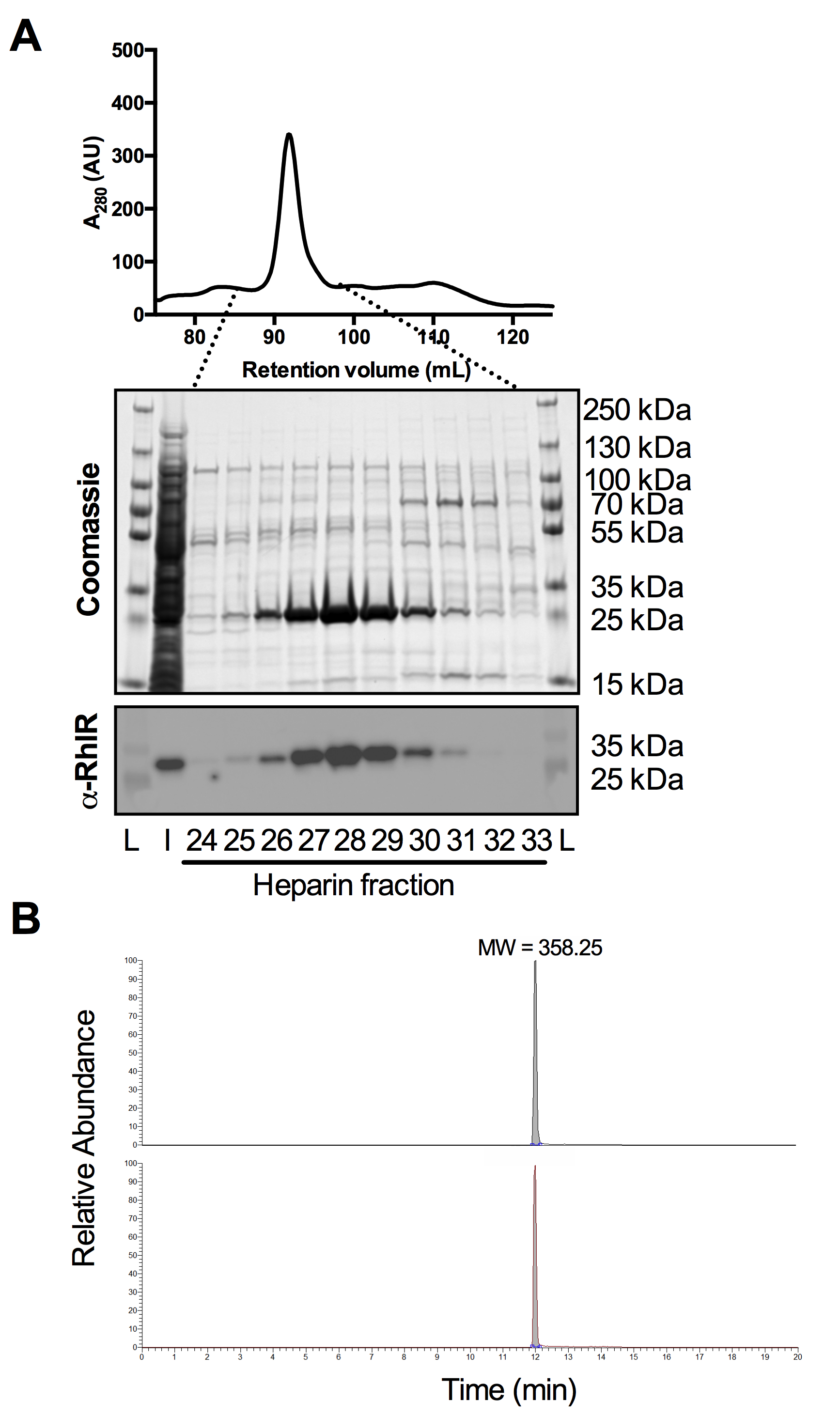

Supplement: S3 Fig — A) Shown are the results from the initial step of purification of RhlR:mBTL by heparin chromatography. Top: UV280 chromatogram of the peak fractions from the heparin column. AU denotes arbitrary units. Center: Coomassie-stained gel analysis of peak fractions 24–33 (shown by the dotted lines in the top panel). 1% total volume from peak fractions was loaded in each lane. Bottom: Immunoblot of peak fractions 24–33 using an anti-RhlR antibody. Molecular weight markers are designated to the right of the gel. “L” and “I” denote ladder and input, respectively. B) Extracted ion chromatogram of 1 μM mBTL control sample (top) and the mBTL released from 2 μg of purified RhlR protein (bottom, see pooled fractions from Fig 2B of the main text). The observed and known molecular weights of mBTL are identical. (TIFF) [file ppat.1007820.s003.tiff]

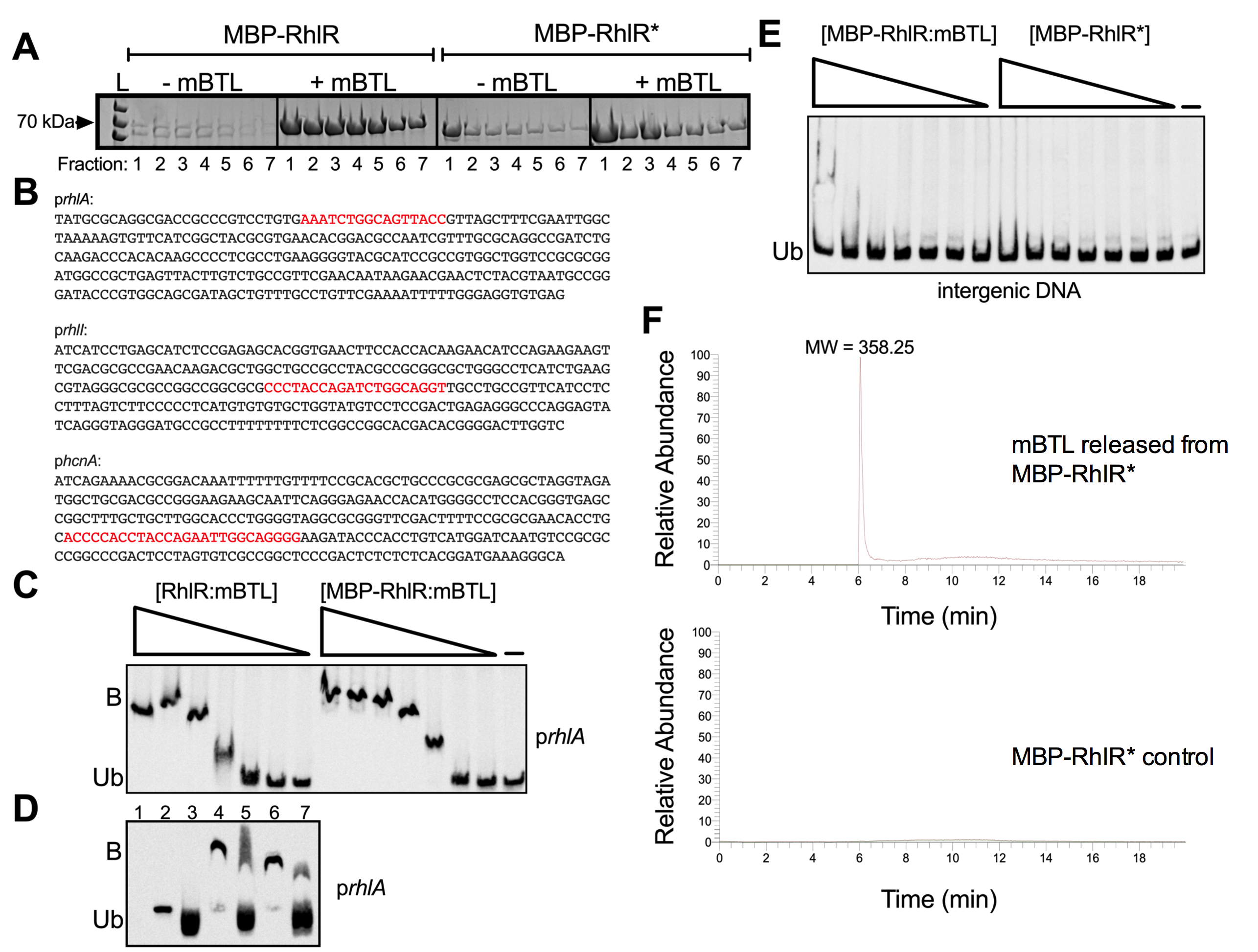

Supplement: S4 Fig — A) The soluble fractions from lysed E. coli cells expressing MBP-RhlR or MBP-RhlR* that had been grown in the presence or absence of mBTL were incubated, in bulk, with amylose resin and eluted with 10 mM maltose in lysis buffer (see Materials and Methods). Seven 1 mL fractions were collected and 1% of the total volume of each fraction was subjected to SDS PAGE analysis. “L” denotes ladder and the 70 kDa band is designated. B) DNA sequences from -300 to -1 bp of the rhlA, rhlI, and hcnA promoters. Red sequences show the rhl-boxes. C) Electrophoretic mobility gel shift showing the 300 bp biotin-labeled rhlA promoter sequence incubated with decreasing concentrations of RhlR:mBTL and MBP-RhlR:mBTL. “Ub” and “B” denote unbound DNA and DNA bound to protein, respectively. The probe DNA was used at 30 ng with 500, 200, 100, 50, 30, 20, and 10 ng of the specified protein going from left to right on the gel. The right-most lane shows the no protein control (designated by the dash). D) Electrophoretic mobility gel shift showing the 300 bp rhlA promoter sequence labeled with biotin with or without the identical unlabeled competitor DNA. Lanes are as follows: 1) unlabeled competitor DNA alone, 2) labeled DNA alone, 3) labeled DNA and unlabeled competitor DNA, 4) labeled DNA and RhlR:mBTL, 5) labeled DNA, RhlR:mBTL, and unlabeled competitor DNA, 6) labeled DNA and MBP-RhlR:mBTL, and 7) labeled DNA, MBP-RhlR:mBTL, and unlabeled competitor DNA. The unbound biotin-labeled DNA band spreads out when it is combined with the 100-fold excess unbound unlabeled competitor DNA. This feature makes the unbound band appear thicker than when no competitor DNA is present. “Ub” and “B” denote unbound DNA and DNA bound to protein, respectively. The labeled probe DNA was used at 30 ng and unlabeled probe DNA was used in 100-fold excess. 200 ng of the different proteins were used. E) Electrophoretic mobility gel shift showing a biotin-labeled 300 bp fragment of intergenic control DNA with different [file ppat.1007820.s004.tiff]

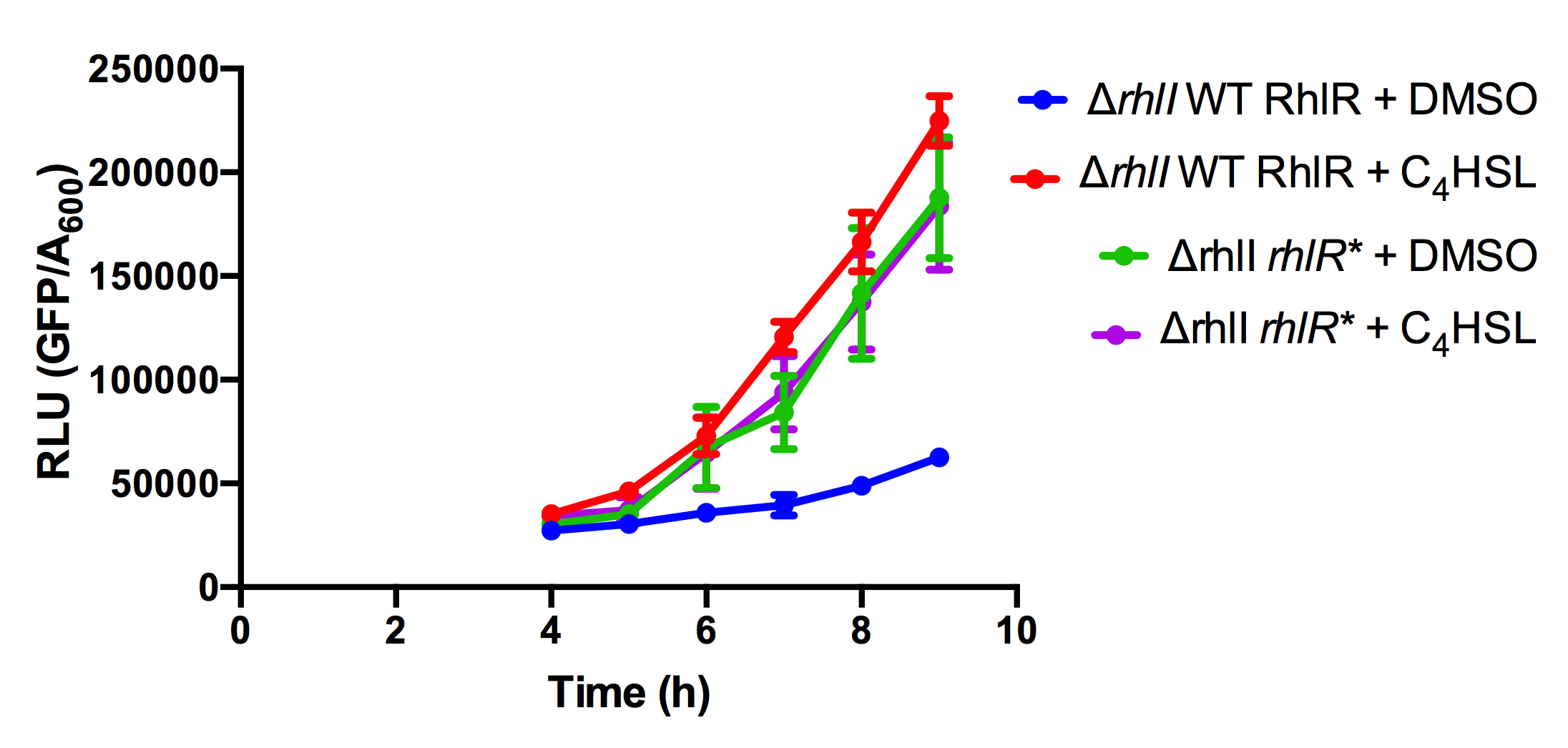

Supplement: S5 Fig — Shown are 9 h time-courses of RhlR- and RhlR*-dependent activation of expression of a prhlA-mNeonGreen transcriptional fusion in ΔrhlI P. aeruginosa in the presence of 10 μM C4HSL or 1% DMSO. Data depict the mean of 3 biological replicates. Two technical replicates were performed for each biological replicate. Error bars depict the standard error of the mean of the biological replicates. (TIFF) [file ppat.1007820.s005.tiff]

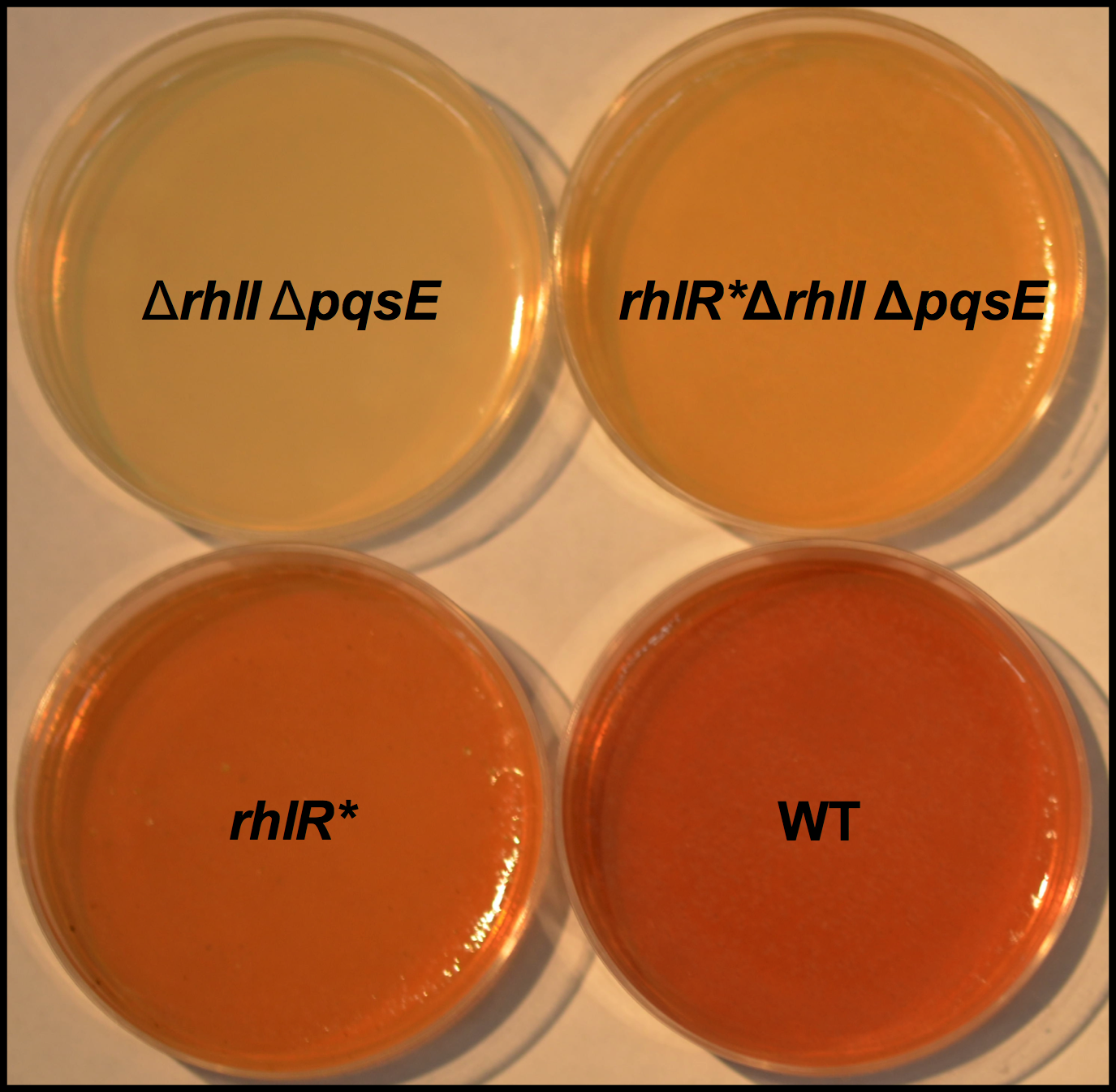

Supplement: S6 Fig — Pyocyanin production phenotypes of the designated P. aeruginosa strains are shown following growth for 48 hours on PGS medium. A 10 μL aliquot of stationary phase P. aeruginosa was spotted onto each plate and the sample was spread evenly over the entire agar surface. There were no C. elegans added to the plates. Red coloration is indicative of pyocyanin production. Images are representative of 3 biological replicates and 2 technical replicates for each biological replicate. (TIFF) [file ppat.1007820.s006.tiff]

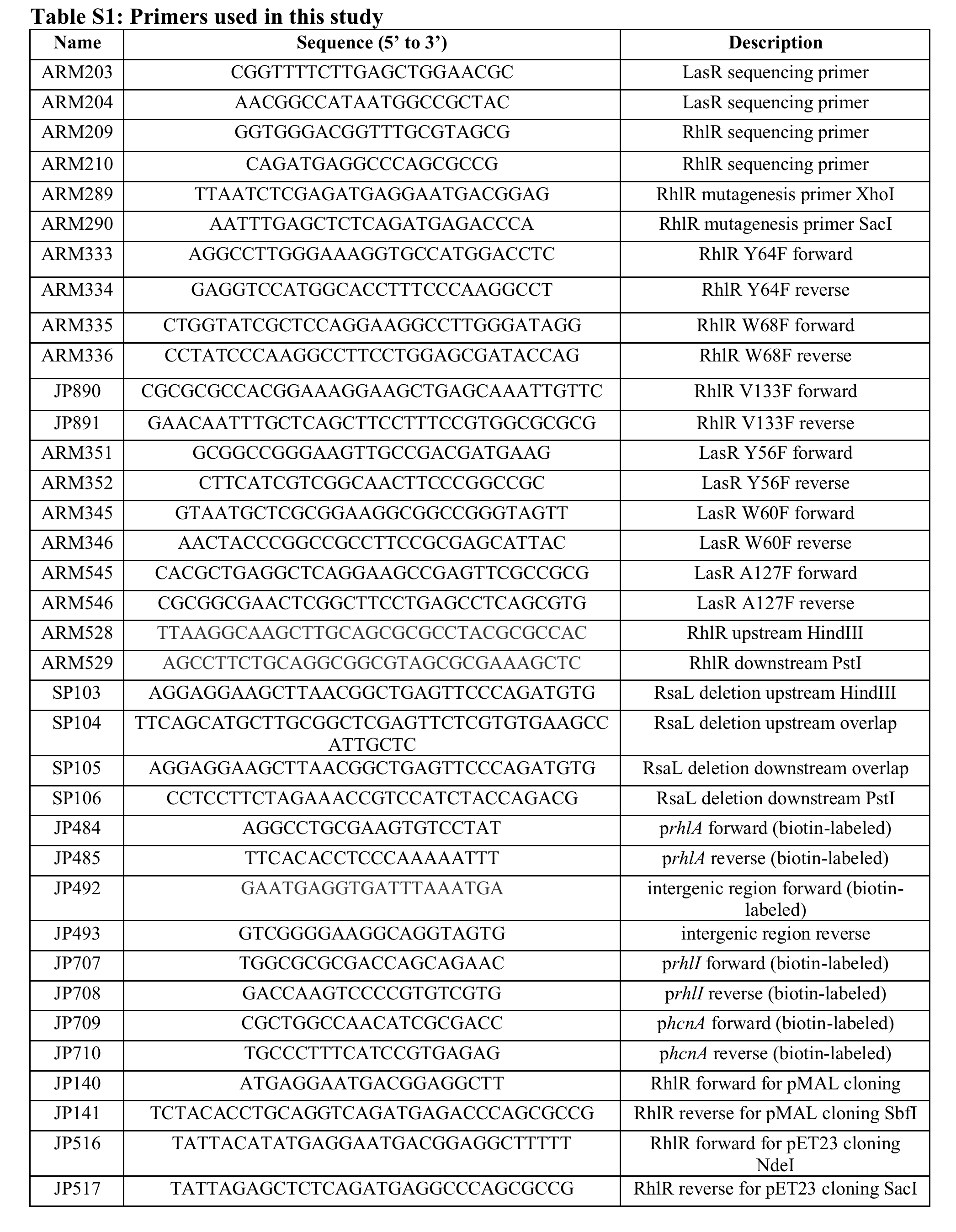

Supplement: S1 Table — This table lists the names, sequences (5’ to 3’), and uses of all primers employed in this study. (ZIP) [file ppat.1007820.s007.zip › Table S1.tiff]

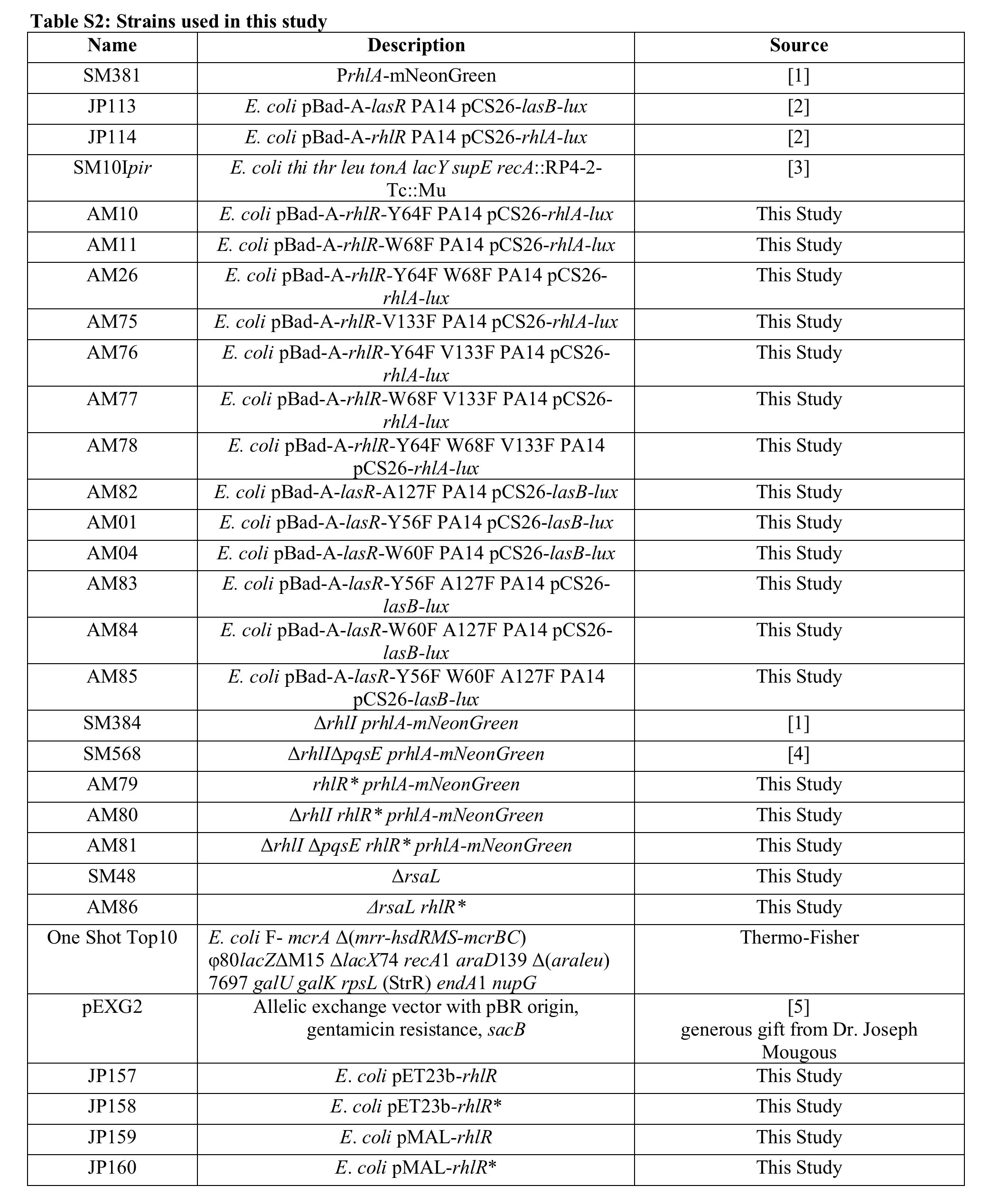

Supplement: S2 Table — This table lists the names, sources, and genotypes of all strains used in this study. (ZIP) [file ppat.1007820.s008.zip › Table S2.tiff]
